# Multi-Tissue DNA Methylation Remodeling at Mitochondrial Quality Control Genes According to Diet in Rat Aging Models

**DOI:** 10.3390/nu12020460

**Published:** 2020-02-12

**Authors:** Patrizia D’Aquila, Francesco De Rango, Francesco Guarasci, Maurizio Mandalà, Andrea Corsonello, Dina Bellizzi, Giuseppe Passarino

**Affiliations:** 1 Department of Biology, Ecology and Earth Sciences, University of Calabria, 87036 Rende, Italy; m.mandala@unical.it (M.M.); dina.bellizzi@unical.it (D.B.); g.passarino@unical.it (G.P.); 2 Italian National Research Center on Aging, 87100 Cosenza, Italy; guarasci.francesco@gmail.com (F.G.); a.corsonello@inrca.it (A.C.)

**Keywords:** DNA methylation, mitochondrial dynamics, mitochondrial fission, mitochondrial fusion, mitochondrial biogenesis, aging, nutrition

## Abstract

An adequate mitochondrial quality control system ensures the maintenance of a healthy mitochondrial pool so as to slow down the progressive accumulation of damage affecting mitochondrial function during aging and diseases. The amount and quality of nutrients availability were demonstrated to induce a process of bioenergetics adaptation by influencing the above system via epigenetic modifications. Here, we analyzed DNA samples from differently-aged rats fed a standard or low-calorie diet to evaluate tissue-specific changes in DNA methylation of CpG sites falling within *Polg*, *Polg2*, *Tfam*, *Fis1*, and *Opa1* genes. We found significant changes according to age and tissue type and the administration of the low-calorie diet is responsible for a prevalent increase in DNA methylation levels. Particularly, this increase was more appreciable when this diet was administered during adulthood and at old age. Regression analysis demonstrated that DNA methylation patterns of the analyzed genes were negatively correlated with their expression levels. Data we obtained provide the first evidence about changes in DNA methylation patterns of genes involved in the mitochondrial biogenesis in response to specific diets and demonstrated that epigenetic modifications are involved in the modulation of mitochondrial dynamics driven by age and nutrition.

## 1. Introduction

The progressive accumulation of functional impairments in mitochondrial function has been widely recognized as one of the main hallmarks of aging and age-related diseases [1,2]. The age-related mitochondrial decline is partially due to a failure in the fine-tune coordination of mitochondrial turnover mechanisms that regulate the morphology, the size and the number of functional mitochondria under a wide variety of both physiological and non-physiological conditions [3,4,5]. These mechanisms include the increase of mitochondrial mass (biogenesis), the division of a mitochondrion in two or more daughter organelles (fission), the merging of healthy and damaged mitochondria (fusion) and the selective clearance of damaged mitochondria via the autophagic pathway (mitophagy) [4,6,7]. The accumulation of dysfunctional mitochondria leads the drop of mitochondrial energy production, the loss of mitochondrial membrane potential, the burst of damage to cellular macromolecules by mitochondrial Reactive Oxygen Species (ROS), the dysregulation of calcium homeostasis, and alteration in cell growth and death [8]. It follows that the progressive loss in mitochondrial quality control system is decisive in driving toward the overall physiological unbalance typical of aging process [4,5].

Moreover, studies on mitochondrial integrity and function have revealed that mitochondrial architecture undergoes to a process of bioenergetics adaptation in response to particular conditions, including changes in energy demand or nutrient supply, to prevent or maximize Adenosine triphosphate (ATP) synthesis [9,10]. Nutrient excess was demonstrated to induce a shift towards mitochondrial fission and to inhibit mitophagy, thus, increasing ROS levels and the pool of dysfunctional mitochondria [9,11,12,13,14]. Conversely, nutrient scarcity was found to be associated with mitochondrial fusion and elongation [14,15]. In addition, lipid oversupply can alter mitochondrial dynamics [16]. In particular, in skeletal muscle, lipid oversupply negatively influences the expression of genes involved in mitochondrial biogenesis such as members of mitochondrial complex I and II as well as peroxisome proliferator-activated receptor gamma coactivator 1-alpha (PGC1α) and peroxisome proliferator-activated receptor gamma coactivator 1-beta (PGC1β), key regulators of mitochondrial DNA replication [17,18,19]. This evidence was recently confirmed by Xu et al., who reported a decrease in mitochondrial number, ATP synthesis and mitochondrial membrane potential in skeletal muscle of 8- and 16-week old mice fed a high-fat diet [20]. Furthermore, a high-fat diet has been demonstrated to downregulate the protein levels of Sirtuin 3 (SIRT3), deacetylases that regulate not only mitochondrial metabolism, but also a number of regulators of mitochondrial function [21]. In vitro experiments also demonstrated that C2C12 cells treated with ceramide and palmitic acid showed increased mitochondrial fission, loss in ATP synthesis and increased oxidative stress [22,23]. Moreover, it was demonstrated the shift towards mitochondrial biogenesis in the skeletal muscle of mice fed with a high-fat diet supplemented by Omega-3 polyunsaturated fatty acids (PUFAs) such as eicosapentaenoic (EPA) docosahexaenoic (DHA) acids. Mitochondrial abundance, associated to the upregulation of PGC1α and Nuclear Respiratory Factor 1 (NRF1) transcription factors resulted also in the increase of fatty acid oxidation, suggesting the favorable effect of this diet in counteracting lipotoxicity and the onset of insulin resistance [24,25]. Overall promotion of mitochondrial fusion by omega-3PUFAs was also widely demonstrated [26,27,28,29]. In particular, Casanova et al. (2014) demonstrated that DHA also influences mitochondrial morphology since cultured myocytes displayed giant and elongated mitochondria and the downregulation of fission-promoting genes, including Fis1 and Drp1 [29]. Consistent results were observed in skeletal muscle and liver of Wistar rats [27,28].

If it has been largely demonstrated that caloric restriction (CR), namely the reduction of caloric intake (from 10 to 40%), exerts beneficial effects on the extension of lifespan in numerous model organisms; less clear is the evidence about its putative role on mitochondrial dynamics, likely because of the heterogeneity of the degree of energy restriction, the duration of treatment and the tissue type in both humans and model organisms. Alongside studies demonstrating that CR promotes biogenesis, many others could not find any significant correlation between the diet and mitochondrial mass, fusion and fission [30,31,32,33,34,35,36,37].

Calorie restriction, micronutrients supplementation as well as fasting and fasting-mimicking diets were recently demonstrated being responsible for the massive intracellular reprogramming network occurring in aging across a wide variety of living organisms. This reprogramming is mainly mediated by epigenetic mechanisms and result in significant structural changes in chromatin that ultimately result in the alteration of gene expression [38,39,40,41,42,43].

Given the pivotal role of mitochondria both in energy metabolism and in producing substrates for DNA methylation and histone modifications as well as the ability of nutrition to induce epigenetic remodeling, we explored in rats whether changes in eating habit during life were able to modulate the methylation status of candidate genes involved in mitochondrial fusion/fission and biogenesis processes. To this purpose, we searched for tissue-specific differences in the methylation status of Cytosine-phosphate-Guanine (CpG) islands falling within pivotal genes previously found to be involved in mitochondrial biogenesis, fusion and fission (*Polg*, *Polg2*, *Tfam*, *Fis1* and *Opa1*) in DNA samples from differently aged rats fed with standard or low-calorie diet.

## 2. Materials and Methods

### 2.1. Animals

Experiments were performed on Sprague-Dawley rats breeding locally in the animal care facility of the University of Calabria (Italy). Animals (*n* = 3 for experimental condition) were housed in light (12:12 h light-dark cycle) and temperature (22 °C) controlled rooms with free access to food (ssniff diet V1535, Ferdinand-Gabriel-Weg 16 D-59494 Soest, German, metabolizable energy 3.057 kcal/kg) and water. The rats were divided into two groups: the first (control group) was fed with standard diet up to 27, 36, and 96 weeks old, the second (treated group) was fed with low-calorie diet (60% of the intake) for a total period of 6 months started at the age of 3 weeks, 12 weeks and 72 weeks.

Water and food intake were recorded every other day while body mass was recorded monthly. Animals were euthanized with inhalation of Diethyl ether followed by cervical transection and immediately sacrificed. All procedures were conducted according to the European Guidelines for the care and use of laboratory animals (Directive 2010/63/EU) and in accordance with Italian law; the study was authorized with authorization number 295/2016-PR by the Ethical committee of the Ministry of Health.

### 2.2. DNA and mRNA Extraction

500 µL of rat peripheral blood was drawn by cardiac puncture and kept on ice in the presence of DNA extraction buffer (10 mM Sodium chloride (NaCl), 20 mM Tris-Buffer (Tris-HCl) pH 8.0, 1 mM Ethylenediaminetetraacetic acid (EDTA). Heart, liver, kidney, brain, lung and a vessel (mesenteric artery) were excised, placed in cold 4-(2-hydroxyethyl)-1-piperazineethanesulfonic acid (HEPES) physiological saline solution (HEPES-PSS pH7.4, 141.8 mM NaCl, 4.7 mM Potassium chloride (KCl), 1.7 mM Magnesium sulfate (MgSO_4_), 0.5 mM EDTA, 2.8 mM Calcium chloride (CaCl_2_), 10 mM HEPES, 1.2 mM Potassium phosphate monobasic (KH_2_PO_4_), 5 mM Glucose), weighed and thoroughly homogenized in presence of DNA extraction buffer. Then, 10% sodium dodecyl sulfate (SDS) and 10 mg/mL of proteinase K were added to all samples, which were then vigorously vortexed and incubated at 37 °C for 48 h with periodical mixing. Genomic DNA was obtained by phenol/chloroform purification.

Total RNA from peripheral blood was obtained from 500 µL of samples by using the QIAamp RNA Blood Mini kit, according to manufacturer’s protocol. 50 mg of frozen heart, liver and kidney were excised and homogenized in buffer RTL and total RNA was purified using RNeasy Mini Kit (Qiagen, Hilden, Germany) according to manufacturer’s recommendations and RNA samples were treated with DNA-free DNase to remove any residual genomic DNA contamination.

The DNA and RNA concentration and purity were determined spectrophotometrically and purity of the sample evaluated using the 260/280 nm absorbance ratio.

### 2.3. Primer Design for EpiTYPER Assay

Polymerase chain reaction (PCR) primers for the genes of interest were designed using Sequenom’s EpiDesigner (Sequenom, Inc., San Diego, CA, USA) software (Table S1). They do not contain CpGs, amplify both methylated and unmethylated sequences equally, and, due to degradation of DNA during bisulfite conversion, delimit amplicon of size below 300 bp to increase the amplification success rate, thus, covering as many CpGs as possible. For this reason, the regions of interest of *Opa1* and *Fis1* genes were amplified in adjacent and overlapping three and two amplicons, respectively.

A T7-promoter tag (CAGTAATACGACTCACTATAGGGAGAAGGCT) was added to the reverse primers for the in vitro T7 transcription and a 10-mer tag sequence (AGGAAGAGAG) was added to the forward primers to balance the PCR primer length.

### 2.4. Bisulfite Treatment and PCR Conditions

Bisulfite conversion of each DNA sample was performed using the EZ-96 DNA Methylation-Gold kit (Zymo Research, Euroclone, Milan, Italy), according to the manufacturer’s protocol. Briefly, 1 μg of genomic DNA was added to 130 μL of CT conversion reagent in a final volume of 150 μL. The mix was incubated at 98 °C for 10 min and, successively, at 64 °C for 2.5 h. After adding 400 μL of M-binding buffer to the wells of the silicon-A binding plate, each sample was loaded into the wells and centrifuged at 3000× *g* for 5 min. After adding 400 μL of M-wash buffer to the wells and centrifugation at 3000× *g* for 5 min, 200 μL of M-desulfonation buffer was added to each well and incubated at room temperature for 20 min. Then, the solution was removed by centrifugation at 3000× *g* for 5 min and the wells were washed twice with 400 μL of M-wash buffer. Deaminated DNA was eluted in 30 μL of M-elution buffer. The PCRs were carried out in a total volume of 5 μL using 1 μL of bisulfite-treated DNA, EpiTaq PCR buffer 1X 0.4 μM of each primer, 0.3 mM Deoxynucleotide (dNTP) mixture, 2.5 mM of MgCl_2_, and 0.005 U TaKaRa EpiTaq hot-start (HS) (TaKaRa, Diatech Lab Line, Milan, Italy). The thermal profile used for the reaction included a 4-min heat activation of the enzyme at 95 °C, followed by 45 cycles of denaturation at 94 °C for 20 s, annealing at optimal temperature for each primer pair (Table S1) for 30 s, extension at 72 °C for 1 min, then one cycle at 72 °C for 3 min. 0.5 μL of each PCR product was electrophoresed on 1.5% agarose gel to confirm successful PCR and amplification specificity.

### 2.5. Dephosphorylation of Unincorporated Deoxynucleosidetriphosphates and in vitro Transcription and RNaseA Cleavage

Unincorporated dNTPs in the amplification products were dephosphorylated by adding 1.7 μL DNase free water and 0.3 μL (0.5 U) shrimp alkaline phosphatase (SAP) (Sequenom, Inc., San Diego, CA, USA). Each reaction was incubated at 37 °C for 40 min, and SAP was then heat inactivated at 85 °C for 5 min. Subsequently, samples were incubated at 37 °C for 3 h with 5 μL of T-cleavage reaction mix (Sequenom), containing 3.21 μL RNAse-free water, 0.89 μL 5X T7 polymerase buffer, 0.22 μL T-cleavage mix, 0.22 μL 100 mM DTT, 0.40 μL T7 RNA polymerase and 0.06 μL RNase A, for concurrent in vitro transcription and base-specific cleavage. The samples of cleaved fragments were then diluted with 20 μL of water. Conditioning of the cleavage reaction was carried out by adding 6 mg of clean resin.

### 2.6. Mass Spectrometry

10 nl of the resultant cleavage reactions were spotted onto silicon matrix preloaded chips (Spectro-CHIP; Sequenom) using the MassARRAY nanodispenser (Sequenom) and analyzed using the MassARRAY Compact System matrix-assisted laser desorption/ionization-time-of-flight mass spectrometer (MALDI-TOF) (Sequenom). The spectra’s methylation ratios were calculated using EPITYPER software v1.0 (Sequenom). The method yields quantitative results for each of the sequence-defined analytic units referred as CpG units, which may contain either one individual CpG site or an aggregate of CpG sites. Triplicate independent analyses from sodium bisulfite-treated DNA samples were undertaken. The effectiveness of the entire experimental procedure was assessed by analyzing as control CpGenome Universal Unmethylated DNA (Chemicon, Millipore, Nuremberg, Germany) and CpGenome Universal Methylated DNA (Chemicon) in serial mixtures of methylated and unmethylated products, with 10% methylation increments. Data quality control and filtering were carried out by the removal of the CpG dinucleotides whose measurement success rate was <90%. Poor-quality and non-valuable data for the quantitative methylation of each CpG unit measured by MALDI-TOF-MS were excluded.

### 2.7. Expression Profile Analysis of rat Polg, Polg2, Tfam, Fis1, and Opa1 Genes

Reverse transcriptase-PCRs (RT–PCR) were carried out using the RevertAid RT Kit (Thermo Fisher Scientific, Milan, Italy). First, an RT mix including 500 ng of total RNA and 1 μL of Oligo(dT)18 primers was preheated at 65 °C for 5 min. Then, the reaction was carried out in a 20 μL final volume containing 1X reaction buffer, 20 U of RiboLock RNase inhibitor, 1 mM of dNTP mix, and 200 U of RevertAid M-MuLV RT reverse transcriptase. The mix was incubated at for 60 min at 42 °C and, successively, at 70 °C for 5 min to inactivate the reverse transcriptase. The cDNAs obtained were then used as a template for real-time PCRs carried out using the SYBR Green qPCR Master Mix (Promega, Milan, Italy) in a StepOne Plus machine (Applied Biosystems, Milan, Italy). Forward and reverse primers were reported in Table S2.

The final PCR mixture (15 µL) contained 1 µL of cDNA, SensiFAST SYBR Hi-ROX Mix 1X (Bioline, London, UK) and 0.2 µM of each primer. The thermal profile used for the reaction included a 2-min heat activation of the enzyme at 95 °C, followed by 35 cycles of denaturation at 95 °C for 15 s and annealing/extension at 60 °C for 60 s, followed by melt analysis ramping at 60–95 °C. All measurements were taken in the log phase of amplification. Negative controls (in which water instead of cDNA was added) were also run in each plate. StepOne Software V 2.0 was used to analyze data. Gene expression values were normalized to Glyceraldehyde-3-phosphate dehydrogenase (GAPDH) gene expression, used as internal control and 27 weeks old rat samples were used as reference values (relative quantification) for the other samples.

### 2.8. Quantification of mtDNA Copy Number

ND1 and actin beta genes, representing either mitochondrial or nuclear DNA, were used in Real-time quantitative PCR reactions to quantify mtDNA copy number. The PCR mixture (15 µL) consisted of 1 µL of DNA (100 ng), SensiFAST SYBR Hi-ROX Mix 1X (Bioline, London, UK) and 0.2 µM of each primer. Forward and reverse primers were reported in Table S2. The thermal profile used for the reaction included a 2-min heat activation of the enzyme at 95 °C, followed by 35 cycles of denaturation at 95 °C for 15 s and annealing/extension at 60 °C for 60 s, followed by melt analysis ramping at 60–95 °C. All measurements were taken in the log phase of amplification. Negative controls (in which water instead of cDNA was added) were also run in each plate.

Standard curves for mtDNA (ND1) and actin beta were generated with normal control rats. Templates in each reaction were diluted 1:100,000, 1:10,000, 1:1000, 1:100, 1:10 and 1:1. Substrate concentrations were calculated according to the standard curves. The copy number of mitochondrial gene ND1 was normalized to the single-copy nuclear gene. Relative mtDNA copy number was calculated and expressed by using the 2−^ΔΔCT^ method [44].

### 2.9. Statistical Analyses

Comparisons between groups were tested using unpaired t-test and one-way analysis of variance (ANOVA) followed by Tukey′s post hoc test. Normality was checked for all data before analysis. Methylation differences of at least 10% and *p*-value of < 0.05 were considered statistically significant. Correlation analyses were performed using Pearson’s correlation coefficient. The strength of the Pearson’s correlation coefficients was identified as weak r ≤ 0.3, moderate r = 0.3 ≤ 0.5 and strong r ≥ 0.5.

All statistical analyses were performed using the R system (version 3.6.1).

## 3. Results

### 3.1. CpG Methylation Levels of Polg, Polg2, Tfam, Fis1, and Opa1 in Tissues from Rats of Different Ages

**Figure 1:**
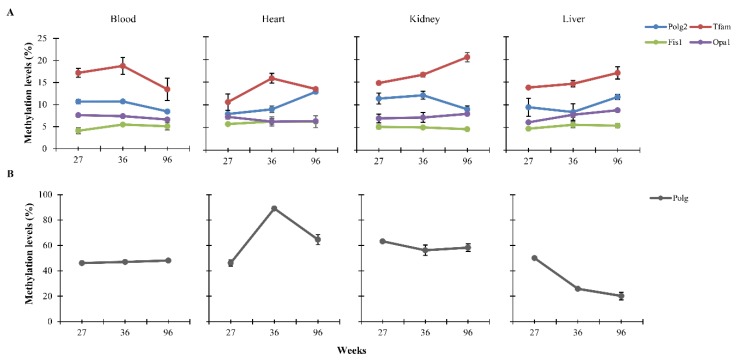
Age-related DNA methylation levels in blood, heart, kidney and liver of differently-aged rats. These levels are reported as arithmetic mean of the CpG sites located within *Polg2, Tfam, Fis1* and *Opa1* (**A**). *Polg* DNA methylation levels are reported in (**B**) because of their different numerical scale.

We assessed age-related changes in methylation of CpG islands located within candidate genes involved in mitochondrial fusion/fission and biogenesis processes. by Sequenom MassARRAY EpiTYPER. Specifically, CpG islands annotations were downloaded from the UCSC genome database. Key genes regulating the above processes, such as *Nrf1*, *Mfn1*, *Mfn2*, *Pgc1-α* and *β*, were found not to have islands, hence, they have not been included in the study. The analysis was carried out in bisulfite-treated DNA samples extracted from four tissues (blood, heart, kidney and liver) of rats of 27, 36 and 96 weeks old. Following stringent quality control criteria (see Materials and Methods), the final dataset included 146 CpG sites organized in single sites or CpG units (Figure S1). We performed a one-way ANOVA followed by Tukey′s post hoc test to investigate changes of DNA methylation levels with age. In Figure 1 for each tissue, age-associated DNA methylation levels are reported as arithmetic mean of the CpG sites of each gene. In particular, *Polg* and *Tfam* in heart showed a gain of DNA methylation in the period of life comprised between 27 and 36 weeks, but a loss of DNA methylation from 36 weeks onwards (*p* < 0.001 and *p* = 0.005, respectively). Moreover, a progressive gain of DNA methylation was observable for *Tfam* in kidney and liver (*p* < 0.001 and *p* = 0.01) and *Opa1* in the liver (*p* = 0.008). *Polg2* in the heart and liver exhibits an increase between 36 and 96 weeks (*p* < 0.001), *Polg* in liver showed a linear decline (*p* < 0.001), while *Polg2* in blood (*p* < 0.001), kidney (*p* = 0.018) and *Tfam* (*p* = 0.037) and *Opa1* in blood (*p* < 0.001) undergo a decrease in DNA methylation levels only after 36 weeks. No changes in DNA methylation according to age were observed for *Polg* in blood and kidney, *Opa1* in blood, heart and kidney and *Fis1* in all tissues analyzed.

### 3.2. Dietary Effects on CpG Methylation Levels of Polg, Polg2, Tfam, Fis1, and Opa1 in Tissues from Rats of Different Ages

**Figure 2:**
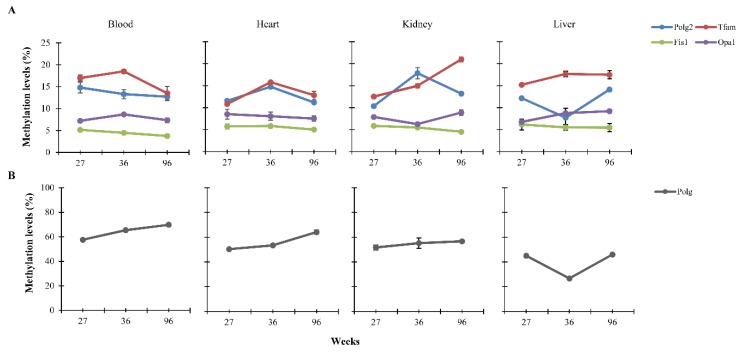
Age-related DNA methylation levels in blood, heart, kidney and liver of differently-aged rats fed low-calorie diet. These levels are reported as arithmetic mean of the CpG sites located within *Polg2*, *Tfam*, *Fis1* and *Opa1* (**A**). *Polg* DNA methylation levels are reported in (**B**) because of their different numerical scale.

In order to investigate nutritional effects during age on DNA methylation status of *Polg*, *Polg2*, *TFAM*, *Fis1* and *Opa1* genes, quantification of CpG methylation levels were carried out in bisulfite-DNA samples extracted from the four tissues of rats of 27, 36 and 96 weeks old fed low-calorie diet for 24 weeks. As shown in Figure 2, a linear gain of DNA methylation was observable for *Polg* in the blood (*p* < 0.001) and heart (*p* < 0.001) and *Tfam* in the kidney (*p* < 0.001). This increase was also observed for *Polg2* in the heart (*p* < 0.001) and kidney (*p* < 0.001) and *Tfam* in the heart (*p* < 0.001) up to 36 weeks of age, and then, a decrease up to 96 weeks occurs. *Tfam* methylation increases in the liver up to 36 weeks, then, remaining constant (*p* = 0.013). Conversely, *Polg* (*p* < 0.001) and *Polg2* (*p* = 0.002) DNA methylation levels decrease in the liver between 27 and 36 weeks and, then, increase from 36 weeks onwards. The only negative trend is observable for *Tfam* (*p* = 0.002) in blood from 36 weeks onwards. No significant differences in DNA methylation levels were observed for *Polg* in the kidney, *Polg2* in the blood and for *Fis1* and *Opa1* in all the tissues analyzed.

**Figure 3:**
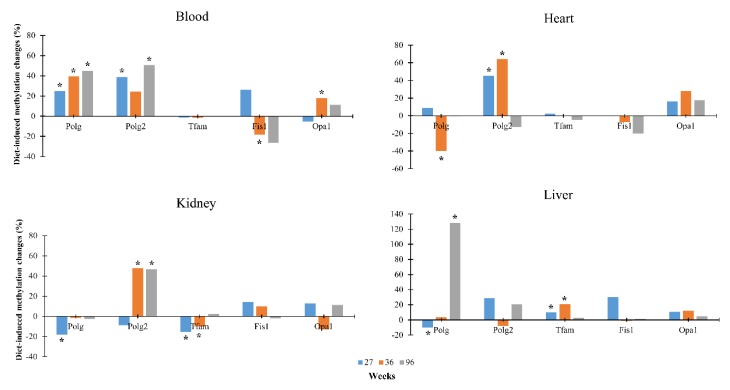
Diet-induced methylation changes in *Polg*, *Polg2*, *Tfam*, *Fis1* and *Opa1* genes in blood, heart, kidney and liver in differently-aged rats. * *p*-value < 0.05 and changes >10%.

To get the endpoint of changes in the DNA methylation in response to the nutritional regime, we compared, for each age, the mean methylation levels between the low-calorie samples with the age-matched controls. We found that diet induces a significant change in DNA methylation levels during aging. The increment and decrement percentage for each gene are shown in Figure 3 and Table S3. *Fis1* and *Opa1* did not show any relevant change.

For clarity, we have reported in Table S4 DNA methylation values of the CpG sites of each gene used to determine the mean values reported in the analyses.

### 3.3. Correlation among Methylation, mRNA Levels and mtDNA Copy Number

To explore the functional relevance of the changes in DNA methylation of the analyzed genes, quantitative real-time PCR assays were carried out to evaluate the expression of *Polg*, *Polg2*, *Tfam*, *Fis1* and *Opa1* in blood, heart, kidney and liver samples from rats of different age and fed standard or low-calorie diet. We observed that *Polg*, *Polg2*, *Tfam* and *Opa1* are differently expressed in both standard or low-calorie diet according to the DNA methylation changes. Conversely, *Fis1* did not exhibit any significant change (Figure S2). Then, we carried out a regression analysis in order to investigate the association between the expression of *Polg*, *Polg2*, *Tfam*, *Fis1* and *Opa1* genes and the corresponding DNA methylation profiles. The analysis revealed an inverse relationship, in most cases statistically significant (Table 1).

The only discrepancy for the *Tfam* gene at 36 weeks in standard-fed conditions (in which a significant positive relationship was observed) was due to the missing correlation between *Tfam* methylation and expression in the heart.

Then, we determined whether DNA methylation levels influenced the mtDNA copy number (Figure S3). Regression analysis did not show a significant correlation except for *Polg2* at standard-fed conditions (96 weeks) and *Polg* and *Polg2* at low-calorie diet (36 weeks) (Table 1).

## 4. Discussion

Understanding the molecular basis of lifespan-extending interventions is one of the most difficult and arduous challenges for researchers. In this frame, epigenetic modifications have emerged as newsworthy features given their peculiar plasticity to undergo more or less consistent changes in response to a wide range of environmental changes, including lifestyle and dietary habits. In particular, nutrient availability can regulate mitochondrial quality control system and dynamics, thus, contributing to the onset of specific physiological and pathological phenotypes. In this study, we aimed at disentangling this aspect, by elucidating changes in DNA methylation status of candidate genes involved in mitochondrial fusion/fission and biogenesis processes according to changes in nutrition habit during life.

To this purpose, we searched for tissue-specific differences in the methylation status of CpG islands falling within candidate genes involved in mitochondrial biogenesis, fusion and fission in DNA samples extracted from differently aged rats fed standard or low-calorie diet. Our findings indicated peculiar DNA methylation patterns for each analyzed gene; thus, typical for each tissue from youth to old age is that the same gene shows different variation trends depending on the tissue under examination except for *Fis1*.

The findings here presented, consistent with literature data, demonstrated that an inverse relationship between DNA methylation and the expression of the associated genes occurs with age, thus providing the functional effects of the observed methylation patterns [45,46]. As demonstrated by bioinformatics analysis, most of the CpG sites undergoing to significant changes overlap with Sp1, YY1, NRF-1 and AP-1, which are methylation-sensitive transcription factors and could regulate the mitochondrial fusion/fission and biogenesis processes. These observations lead us to retain that the progressive impairment of the above processes, widely described during aging process, is mostly thought to be ascribed to their epigenetic regulation during the lifetime according to the specific needs of tissues [47,48,49,50]. Of note, the positive correlation between *Tfam* gene methylation and its expression we observed at 36 weeks seems outlandish. This discrepancy is attributable to the high levels of methylation of the gene in the heart which create a bias in the analysis. As also discussed by Barazzoni et al., likely, the peculiar characteristics of the heart tissue and its contractile activity as well as its age-associated oxidative alteration may unavoidably regulate the molecular mechanisms underlying mitochondrial gene expression [51]. Since the DNA methylation levels have been determined as the mean of the methylation values at the CpG sites of each gene, we are confident that they represent the overall methylation status of the whole region analyzed. The significant correlation between methylation and gene expression confirms our assumption.

While methylation appears to influence gene expression, it does not unequivocally affect the mtDNA copy number since we found this association only in a few experimental conditions. These observations take place in a context of scarce and controversial literature in which a series of factors, including age, degree of cellular and tissue differentiation and type represent just a few of the factor involved in the regulation of mitochondrial DNA copy number. To confirm this, the findings of this paper reveal an association between DNA methylation and mtDNA copy number limited to *Polg2* at 96 weeks and *Polg* and *Polg2* at 36 weeks at standard and low-calorie diet, respectively.

When compared to the standard diet, the administration of the low-calorie diet results responsible for a prevalent increase in DNA methylation levels of the all analyzed genes and tissues [52,53]. In agreement to Sziraki et al., we found that diet induce more appreciable epigenetic changes when the low-calorie diet is administered in adulthood, suggesting that the primary changes during aging are not linear and that they either accelerate or decelerate according to nutrition and, possibly, biological age [54,55,56]. More particularly, in line with other previous studies in mice, at old age, it seems that the low-calorie diet can bring DNA methylation pattern near to the levels of those of the young control subjects, as is evident for *Polg2* in the blood and heart [55,56]. This observation leads us to retain that positive effect of diet in slowing down aging process might be driven, at least in part, by the epigenetic regulation of the methylation of genes in processes relevant for maintaining cellular homeostasis.

The involvement of epigenetics in the regulation of genes involved in mitochondrial fusion/fission and biogenesis processes during lifetime is not specific of rats. Indeed, we recently reported evidence about this in human, wherein individuals of 18–108 years old, displaying different aging phenotypes according to cognitive, functional and psychological parameters, genes involved in fusion and fission dynamics emerged as biomarkers of both chronological and biological aging [57]. Furthermore, this evidence is also in line with our previous results reporting that global DNA methylation status exhibits tissue-specific changes during aging [41]. Although caution is necessary when relating results in animal models with humans, and the correlation between rats and human ages are not unique, the findings of the present and previous studies suggest that the effect of diet on mitochondrial dynamics is more important in the adulthood and at old age.

Results we observed provide further evidence about the positive impact of the low-calorie diet in counteracting aging and suggests for the first time that the epigenetic modification of genes involved in biogenesis might mediate such impact.

## Figures and Tables

**Table 1:** Pearson Correlation (R) between the Level of DNA Methylation, *Polg*, *Polg2*, *Tfam*, *Fis1* and *Opa1* Expression and mtDNA Copy Number. CI: Confidence Interval.

		Standard Diet			Low-Calorie Diet		
Weeks		Pearson′s R Correlation	*p*	95% CI	Pearson′s R Correlation	*p*	95% CI
**36**	Polg methylation-Polg expression	−0.799	0.002	−0.941, −0.415	−0.877	<0.001	−0.965, −0.611
	Polg methylation-mtDNA copy number	−0.549	0.065	−0.854, 0.036	−0.835	<0.001	−0.953, −0.502
	Polg2 methylation-Polg2 expression	−0.258	0.418	−0.725, 0.371	−0.672	0.017	−0.899, −0.160
	Polg2 methylation−mtDNA copy number	−0.090	0.782	−0.631, 0.511	−0.794	0.002	−0.940, −0.405
	Tfam methylation-Tfam expression	0.708	0.010	0.225, 0.911	−0.748	0.005	−0.925, −0.306
	Tfam methylation-mtDNA copy number	−0.473	0.121	−0.823, 0.139	0.140	0.664	−0.472, 0.661
	Fis1 methylation-Fis1 expression	−0.179	0.578	−0.683, 0.440	−0.170	0.598	−0.678, 0.448
	Fis1 methylation-mtDNA copy number	−0.600	0.059	−0.873, −0.039	0.178	0.579	−0.441, 0.682
	Opa1 methylation-Opa1 expression	−0.448	0.144	−0.813, 0.169	−0.404	0.193	−0.794, 0.221
	Opa1 methylation-mtDNA copy number	0.258	0.418	−0.371, 0.725	0.388	0.212	−0.239, 0.787
**96**	Polg methylation-Polg expression	−0.925	<0.001	−0.979, −0.748	−0.857	<0.001	−0.959, −0.558
	Polg methylation-mtDNA copy number	0.114	0.725	−0.492, 0.645	−0.171	0.595	−0.678, 0.447
	Polg2 methylation-Polg2 expression	−0.513	0.088	−0.840, 0.086	0.057	0.859	−0.534, 0.611
	Polg2 methylation-mtDNA copy number	0.774	0.003	0.360, 0.933	0.565	0.056	−0.013, 0.860
	Tfam methylation-Tfam expression	−0.746	0.005	−0.924, −0.302	−0.941	<0.001	−0.984, −0.798
	Tfam methylation-mtDNA copy number	0.565	0.055	−0.012, 0.860	−0.054	0.866	−0.609, 0.536
	Fis1 methylation-Fis1 expression	−0.294	0.354	−0.743, 0.337	−0.294	0.354	−0.743, 0.337
	Fis1 methylation-mtDNA copy number	0.457	0.135	−0.804, 0.194	0.522	0.082	−0.074, 0.843
	Opa1 methylation-Opa1 expression	−0.799	0.002	−0.941, −0.416	−0.358	0.253	−0.773, 0.272
	Opa1 methylation-mtDNA copy number	0.009	0.977	−0.568, 0.580	0.284	0.371	−0.347, 0.738

## Supplementary Materials

The following are available online at https://www.mdpi.com/2072-6643/12/2/460/s1, Figure S1: Nucleotide sequence of the CpG islands and graphical representation of the CpG sites and units, located within *Polg*, *Polg2*, *Tfam*, *Fis1* and *Opa1*, as according to EpiTYPER software. Sequenom CpG sites analyzed are highlighted in red. Figure S2: Expression levels of *Polg*, *Polg2*, *Tfam1*, *Fis1* and *Opa1* measured in blood, heart, kidney and liver according to age and tissues in rats fed standard or low-calorie diet. mRNA levels are reported as the mean of relative quantification values (RQ), measured in three independent triplicate experiments with Standard Error Mean (SEM). Figure S3: Relative quantification of mtDNA copy number in blood, heart, kidney and liver according to age and tissues in rats fed standard or low-calorie diet. Levels are reported as the mean of relative quantification values, measured in three independent triplicate experiments with Standard Error Mean (SEM). Table S1: Nucleotide sequence, amplicon size, annealing temperature and chromosomal localization of the primers used in the DNA methylation analysis. Table S2: Nucleotide sequence and chromosomal localization of the primer pairs used in gene expression and mtDNA copy number analyses. Table S3: DNA methylation of *Polg*, *Polg2*, *Tfam1*, *Fis1* and *Opa1* according to age and tissues in rats fed standard or low-calorie diet. Data represent the mean of DNA methylation values of the CpG sites located within the analyzed genes. SD: Standard Deviation; MD: Methylation Differences between standard or low-calorie diet values. Table S4: DNA methylation values of the CpG sites of each gene according to age and tissues in rats fed standard or low-calorie diet. SD: standard deviation.
