# Erratum: Multi-Tissue DNA Methylation Remodeling at Mitochondrial Quality Control Genes According to Diet in Rat Aging Models

**DOI:** 10.3390/nu12051494

**Published:** 2020-05-20

**Authors:** Patrizia D’Aquila, Francesco De Rango, Francesco Guarasci, Maurizio Mandalà, Andrea Corsonello, Dina Bellizzi, Giuseppe Passarino

**Affiliations:** 1 Department of Biology, Ecology and Earth Sciences, University of Calabria, 87036 Rende, Italy; m.mandala@unical.it (M.M.); dina.bellizzi@unical.it (D.B.); g.passarino@unical.it (G.P.); 2 Italian National Research Center on Aging (IRCCS INRCA), 87100 Cosenza, Italy; guarasci.francesco@gmail.com (F.G.); a.corsonello@inrca.it (A.C.)

The Nutrients Editorial Office would like to update the error in the original published version [1].

The affiliation of the third and fifth author, “Italian National Research Center on Aging, 87100 Cosenza, Italy”, has been changed to “Italian National Research Center on Aging (IRCCS INRCA), 87100, Cosenza, Italy”.

We would like to apologize for any inconvenience caused to the authors and readers by this mistake, stating it does not affect the scientific results. We will update the article, and the original version will remain available on the article webpage.
