# Supplementary material for: Multi-Tissue DNA Methylation Remodeling at Mitochondrial Quality Control Genes According to Diet in Rat Aging Models
Source: Nutrients. 2020 Feb 12;12(2):460. doi: 10.3390/nu12020460 (PMC7071227; doi:10.3390/nu12020460)

Tfam - Transcription factor A, mitochondrial - Chromosome 20: 18593830- 18594066

GGCCTTCCAGCAGAATACTCAGAGGGGCCTG**CG**GCTATGG**CGCG**GCTCAGCAACACCCTTGCCAAA
CTAAAC**CG**GCTCTGCCTAGC**CG**CAGGCTC**CG**CCCCCACTCAGCCC**CG**CCCACTGAA**CG**GTGGGGA
CACACTC**CG**CCTCC**CG**TTTGCCC**CG**CCTCCTGCTGCAGAC**CG**GAAGTCTGGGCCTCCCACAGTGCC
C**CGCGCGCGCG**G**CG**GGCATGATAACAAGCCCCTGGAGTA

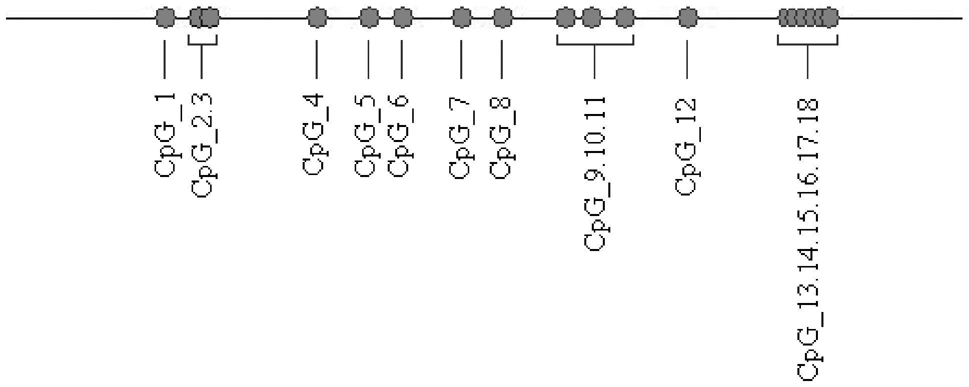

Opa1_Amplicon1 - Mitochondrial dynamin like GTPase - Chromosome 1: 74793424- 74793585

TGGGCCAAGGCATCAAAAATCCTGTTGAA**CG**GGGAGTGGTCAC**CG**GCCAACTCC**CG**TCACTCCTC**C**
GGGTACTTAAACATT**CGCG**AGAATTCTCAAAGAGCTC**CG**GCCTGGCCTGAGGGT**CG**GTCACCTGCA
CATTCAGGAAGTAGTGATGGGTCAACCCC

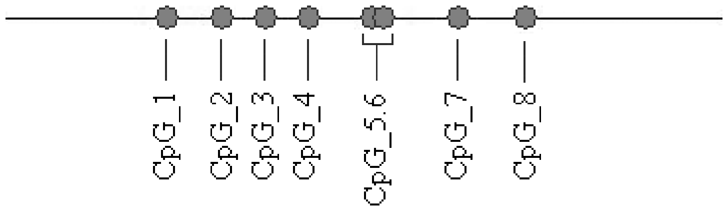

Opa1_Amplicon2 - Mitochondrial dynamin like GTPase - Chromosome 1: 74793558- 74793769

TTCAGGAAGTAGTGATGGGTCAACCCCAC**CG**TCCAGATGGCCCAGAA**CG**TGACATAA**CG**GGGCCAG
GT**CG**CAGGC**CG**CACTTACCAGGCCAGGGCAGCC**CG**ACCTGCT**CG**CCACATCC**CG**CCAG**CG**CAGACC
ACACACAGG**CG**C**CG**AGA**CG**GCCA**CG**GGGGAGCCC**CG**TGT**CG**GCCCCACTC**CG**ACTCCTGCTCCAGC
AATGACCCAGGAA

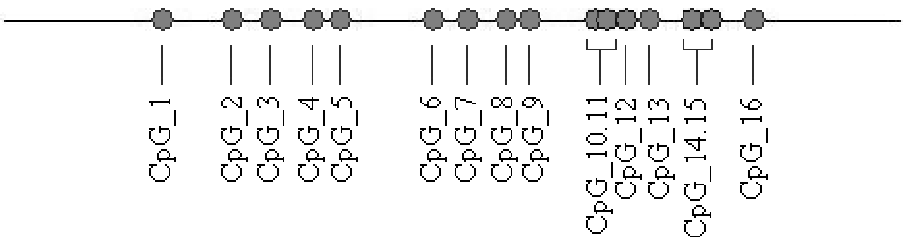

Opa1_Amplicon3 - Mitochondrial dynamin like GTPase - Chromosome 1: 74793743-74794121

CTCCTGCTCCAGCAATGACCCAGGAAG**CG**GCCCTCAGCAACAAGGGCATAGAGCAGC**CG**C**CG**AGTC
CCTCCCAATGG**CG**CATGGACTTC**CG**CAAGATC**CG**GCTGGGCTCC**CG**TAGTCAGTC**CG**TCCC**CG**AAA
TAACCACCTACAGGAA**CG**CTAGGCAGGCTGCACAAATGTGC**CGCG**GATAA**CG**TG**CGCG**TG**CG**CTAG
AGCCTGTGGG**CG**GGACTTGTATAGTGCTCCTCCTCTTGGCTC**CG**CCCCCAAAGTGTC**CG**GGTTGGA
ATTCTGAAAATAAACTTACCCCATTAAGGATTC**CG**GGAGGTCTGGG**CG**GGCTGGG**CGCG**GGAAATC
CCCA**CG**ATTAATATTTACTGGAGCAGCCAATATAAGGAGACCTGTTTC

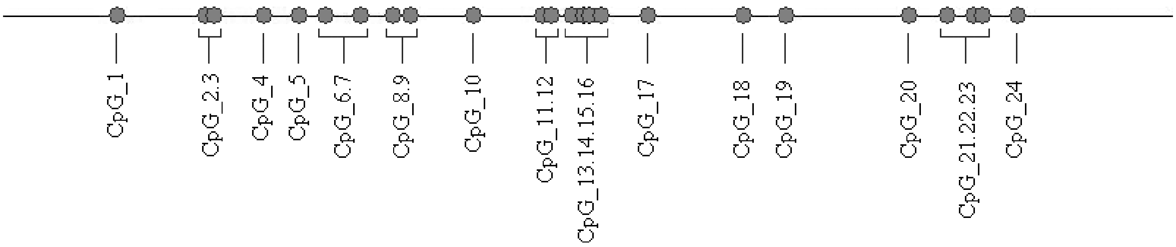

Polg2 - DNA polymerase gamma 2, accessory subunit - Chromosome 10: 94978642-94979091

CCAAAGGTCCAAAGCCTGAGTGGCATC**CG**CTCAGGAGAGCAGC**CG**TGCTGAGCTGCTGTGG**CG**TTC
CGCTGAGAAAGTGCCTCCTC**CG**GCAGAGGTCTACCAG**CG**CCT**CGCG**GGCCAATGCATGCT**CG**GGGT
C**CG**TAGGCTGCTG**CG**TCCCATCTGTGGGACC**CG**CATATC**CG**GACAGCCAGCACCTGCAGGCCCTG**C**
GGCAGGCCCTGGCCA**CG**CCACC**CG**AGTGCAT**CG**CTTC**CG**GACT**CG**GGAACAC**CG**G**CG**CCTCA**CG**TG
GGAAGC**CG**TCTCTATCCTAAACCAGCACCAAAAGCCTAGGG**CG**CAGG**CGCG**G**CG**GAAATGAA**CGCG**
C**CG**CAGACCA**CG**CCCC**CG**GAAGAGCATCACC**CG**CTGCTGGGC**CG**CAGC**CG**CCTTGGG**CG**TATCC**CG**
GCCTC**CGCG**GCTGG**CG**CCAG**CG**TTTGTGGTTGTTTACTACCTTGGGGATTTTC

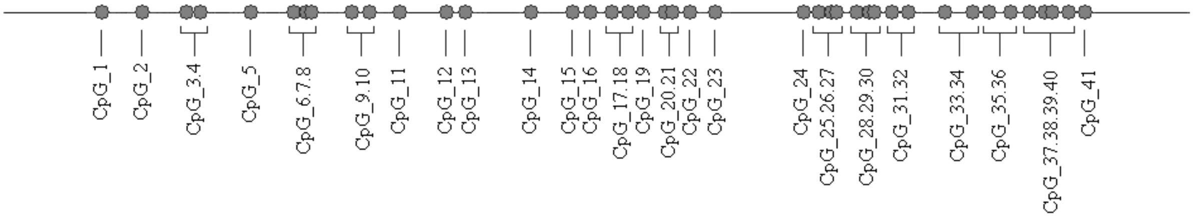

Polg - DNA polymerase gamma, catalytic subunit - Chromosome 1: 141187648-141187891

CAGCATCTGGATGTGCAGAGGGTTGAGC**CG**CAGCTGCCCATTCTCTGAGGAGGGCATTTG**CG**ACTG
CGGCTGCC**CG**T**CG**CTGGGGA**CG**GGGT**CG**AGGA**CG**GAGCTGGAGACCCAG**CG**TCCTGTTGCTGGTAC
TGGCCCTGAGG**CG**ACTTTGG**CG**C**CG**GCCACCTTCTTCCAGAGCAGG**CG**GCTCATGGTTGGTGCAGG
GAGCCCCC**CG**CTGTGAGT**CG**GGACACCTGGCTTTGGGCTCCAGCT

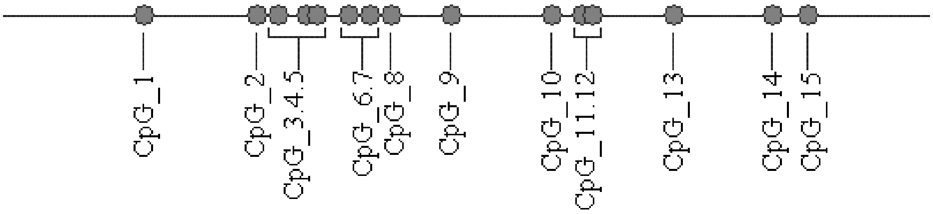

Fis1_Amplicon1 - Fission, mitochondrial 1 - Chromosome 12: 22765212-22765439

TTTCTGACAAAGGACAGTCCCAAGAGGCCAGCCTC**CG**GGTAGAAGAAGTAA**CG**GCAGG**CG**TTACAG
AAAGCA**CG**CTTGGGTCCCTCTGGGAAGCCTGAATTTC**CG**GGTAG**CG**CCTCCTTAGGGGCTTCTCTA
GGAAGT**CG**GCT**CG**ATGCTCTGGAGGACC**CG**GAGCTGATCCACTGGGAAGAG**CG**AGGTATTGAGTCT
G**CG**CAGTAGCTGGCCTTCAAAGAGGGGTG

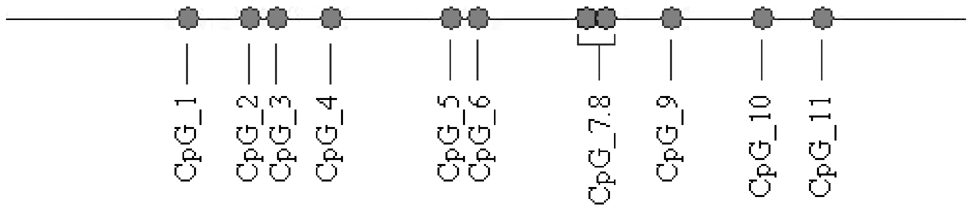

Fis1_Amplicon2 - Fission, mitochondrial 1 - Chromosome 12: 22765324-22765561

CCTCCTTAGGGGCTTCTCTAGGAAGT**CG**GCT**CG**ATGCTCTGGAGGACC**CG**GAGCTGATCCACTGGG
AAGAG**CG**AGGTATTGAGTCTG**CG**CAGTAGCTGGCCTTCAAAGAGGGGTGGGGC**CG**AG**CG**GGGCCAA
GGG**CG**GAG**CG**AGAGGACTG**CG**CTGACCAGTGAA**CGCGCG**CTGCTCTACAAGGGG**CG**GGG**CG**ACACT
AGGAGGG**CG**GGG**CGCG**ACTGGATGCTGGAAAGAGGGTCTC

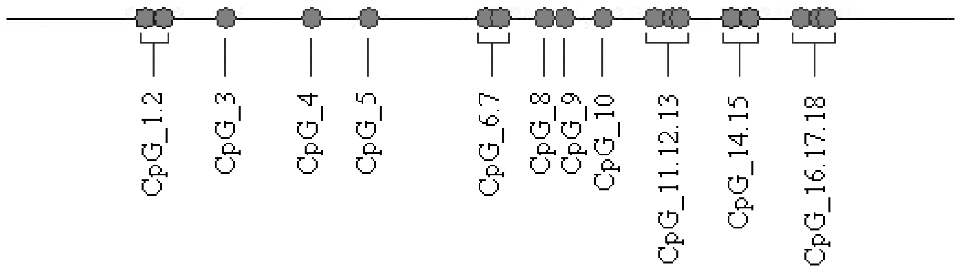

Supplement: Supplementary file 1 [file nutrients-12-00460-s001.zip › nutrients-721651-supplementary/Figure S1.pdf]
