# Supplementary material for: Multi-Tissue DNA Methylation Remodeling at Mitochondrial Quality Control Genes According to Diet in Rat Aging Models
Source: Nutrients. 2020 Feb 12;12(2):460. doi: 10.3390/nu12020460 (PMC7071227; doi:10.3390/nu12020460)
Supplement: Supplementary file 1 [file nutrients-12-00460-s001.zip › nutrients-721651-supplementary/Supplementary Legends.docx]

**Supplementary Legends:**

**Figure S1.** Nucleotide sequence of the CpG islands and graphical representation of the CpG sites and units, located within *Polg*, *Polg2, Tfam, Fis1* and *Opa1*, as according to EpiTYPER software. Sequenom CpG sites analyzed are highlighted in red.

**Figure S2.** Expression levels of *Polg*, *Polg2*, *Tfam1, Fis1* and *Opa1* measured in blood, heart, kidney and liver according to age and tissues in rats fed standard or low-calorie diet. mRNA levels are reported as the mean of relative quantiﬁcation values (RQ), measured in three independent triplicate experiments with Standard Error Mean (SEM).

**Figure S3.** Relative quantification of mtDNA copy number in blood, heart, kidney and liver according to age and tissues in rats fed standard or low-calorie diet. Levels are reported as the mean of relative quantiﬁcation values, measured in three independent triplicate experiments with Standard Error Mean (SEM).

**Table S1.** Nucleotide sequence, amplicon size, annealing temperature, and chromosomal localization of the primers used in the DNA methylation analysis.

**Table S2.** Nucleotide sequence and chromosomal localization of the primer pairs used in gene expression and mtDNA copy number analyses.

**Table S3.** DNA methylation of *Polg*, *Polg2*, *Tfam1, Fis1,* and *Opa1* according to age and tissues in rats fed standard or low-calorie diet. Data represent the main of DNA methylation values of the CpG sites located within the analysed genes. SD: Standard Deviation; MD: Methylation Differences between standard or low-calorie diet values.

**Table S4.** DNA methylation values of the CpG sites of each gene according to age and tissues in rats fed standard or low-calorie diet. SD: standard deviation.
