# Supplementary material for: Multi-Tissue DNA Methylation Remodeling at Mitochondrial Quality Control Genes According to Diet in Rat Aging Models
Source: Nutrients. 2020 Feb 12;12(2):460. doi: 10.3390/nu12020460 (PMC7071227; doi:10.3390/nu12020460)
Supplement: Supplementary file 1 [file nutrients-12-00460-s001.zip › nutrients-721651-supplementary/Table S1.docx]

| **Gene** | **Primer Sequence** | **Amplicon (bp)** | **CpGs analyzed** | **Annealing temperature (°C)** | **Amplicon chromosome localization** | **Target** |
| --- | --- | --- | --- | --- | --- | --- |
| Polg | For: TAGTATTTGGATGTGTAGAGGGTTGA  Rev: AACTAAAACCCAAAACCAAATATCC | 243 | 15 | 60 | Chr 1: 141187648-141187891 | Exon 1 reverse strand |
| Polg2 | For: TTAAAGGTTTAAAGTTTGAGTGGTATT  For: AAAAATCCCCAAAATAATAAACAAC | 449 | 41 | 60 | Chr 10: 94978642-94979091 | Exon 1 reverse strand |
| Tfam | For: GGTTTTTTAGTAGAATATTTAGAGGGG  Rev: TACTCCAAAAACTTATTATCATACCC | 237 | 18 | 60 | Chr 20: 18593830-18594066 | 5’ Upstream region forward strand |
| Fis1 Amplicon1 | For: TTTTTGATAAAGGATAGTTTTAAGAGGTT  Rev: CACCCCTCTTTAAAAACCAACTACT | 227 | 11 | 60 | Chr 12: 22765212-22765439 | Exon 1 reverse strand |
| Fis1 Amplicon2 | For: TTTTTTTAGGGGTTTTTTTAGGAAG  Rev: AAAACCCTCTTTCCAACATCCAAT | 238 | 18 | 60 | Chr 12: 22765324-22765561 | 5’ Upstream region reverse strand |
| Opa1 Amplicon1 | For: TGGGTTAAGGTATTAAAAATTTTGTTG  Rev: AAAATTAACCCATCACTACTTCCTAAA | 161 | 8 | 60 | Chr 1: 74793424-74793585 | Intron 1-2  reverse strand |
| Opa1 Amplicon2 | For: TTTAGGAAGTAGTGATGGGTTAATTT  Rev: TTCCTAAATCATTACTAAAACAAAAATC | 211 | 16 | 60 | Chr 1: 74793558-74793769 | Intron 1-2  reverse strand |
| Opa1 Amplicon3 | For: TTTTTGTTTTAGTAATGATTTAGGAAG  Rev: AAAACAAATCTCCTTATATTAACTACTCC | 378 | 24 | 60 | Chr 1: 74793743-74794121 | Exon 1 reverse strand |

**Table S1.** Nucleotide sequence (5’→3’), amplicon size, annealing temperature, and chromosomal localization of the primers used in the DNA methylation analysis.
