# Supplementary material for: Multi-Tissue DNA Methylation Remodeling at Mitochondrial Quality Control Genes According to Diet in Rat Aging Models
Source: Nutrients. 2020 Feb 12;12(2):460. doi: 10.3390/nu12020460 (PMC7071227; doi:10.3390/nu12020460)
Supplement: Supplementary file 1 [file nutrients-12-00460-s001.zip › nutrients-721651-supplementary/Table S2.docx]

**Table S2.** Nucleotide sequence (5’→3’**)** and chromosomal localization of the primer pairs used in gene expression and mtDNA copy number analyses.

| Polg | For AGTTCAACCACAGGCTCTC  Rev ACCTCCCACTTCTTCCATC | Chr 1: 1411766-141174869 (reverse strand) |
| --- | --- | --- |
| Polg2 | For CCCTTGGAGACTGAACACAC  Rev TCCACCTGCCTCACATTCAC | Chr 10: 94979257-94979102 (reverse strand) |
| Tfam | For ACGCCTAAAGAAGAAAGCAC  Rev CTGACTCATCCTTAGCCTCC | Chr 20: 18597409- 18598747  (forward strand) |
| Fis1 | For CCTGCCGTTACTTCTTCTACC  Rev TCATCCCTTACCACGCAACC | Chr 12: 22765269- 22765151  (reverse strand) |
| Opa1 | For TGACAAAGGCATCCACCAC  Rev TCTCCAACCACAACAACCC | Chr 11: 74769143- 74764670 (reverse strand) |
| ND1 | For GGCTACATACAATTACGCAAAG  Rev TAGAATGGAGTAGACCGAAAG | mtDNA: 2824-3090 |
| Actb | For ATCCGTAAAGACCTCTATGCCAACA  Rev GGCTACAACTACAGGGCTGACCAC | Chr 12: 13718368- 13718544  (forward strand) |
