# Supplementary material for: Multi-Tissue DNA Methylation Remodeling at Mitochondrial Quality Control Genes According to Diet in Rat Aging Models
Source: Nutrients. 2020 Feb 12;12(2):460. doi: 10.3390/nu12020460 (PMC7071227; doi:10.3390/nu12020460)
Supplement: Supplementary file 1 [file nutrients-12-00460-s001.zip › nutrients-721651-supplementary/Table S3.docx]

**Table S3.** DNA methylation of *Polg*, *Polg2*, *Tfam1, Fis1,* and *Opa1* according to age and tissues in rats fed standard or low-calorie diet. Data represent the main of DNA methylation values of the CpG sites located within the analysed genes. SD: Standard Deviation; MD: Methylation Differences between standard or low-calorie diet values.

| **Tissue** |  | **27 weeks** | | | | **36 weeks** | | | | **96 weeks** | | | |
| --- | --- | --- | --- | --- | --- | --- | --- | --- | --- | --- | --- | --- | --- |
|  |  | **Standard diet** | **Low-calorie diet** |  |  | **Standard diet** | **Low-calorie diet** |  |  | **Standard diet** | **Low-calorie diet** |  |  |
|  |  | **Mean (SD)** | **Mean (SD)** | **MD (%)** | **p-values** | **Mean (SD)** | **Mean (SD)** | **MD (%)** | **p-values** | **Mean (SD)** | **Mean (SD)** | **MD (%)** | **p-values** |
| **Blood** | **Polg** | 0.461 (.003) | 0.575 (.005) | 25 | 0.000 | 0.470 (.006) | 0.654 (.011) | 39 | 0.000 | 0.481 (.007) | 0.698 (.012) | 45 | 0.000 |
|  | **Polg2** | 0.107 (.006) | 0.148 (.012) | 39 | 0.006 | 0.107 (.001) | 0.133 (.011) | 24 | 0.050 | 0.084 (.001) | 0.127 (.009) | 51 | 0.014 |
|  | **Tfam** | 0.172 (.010) | 0.170 (.007) | -1 | 0.754 | 0.188 (.019) | 0.185 (.005) | -1 | 0.827 | 0.135 (.025) | 0.135 (.016) | 0 | 0.985 |
|  | **Fis1** | 0.041 (.007) | 0.051 (.004) | 26 | 0.086 | 0.055 (.003) | 0.045 (.003) | -18 | 0.011 | 0.051 (.008) | 0.037 (.004) | -26 | 0.064 |
|  | **Opa1** | 0.076 (.002) | 0.072 (.001) | -5 | 0.036 | 0.074 (.001) | 0.087 (.002) | 18 | 0.000 | 0.066 (.002) | 0.073 (.006) | 11 | 0.093 |
|  |  |  |  |  |  |  |  |  |  |  |  |  |  |
| **Heart** | **Polg** | 0.462 (.025) | 0.503 (.012) | 9 | 0.064 | 0.891 (.015) | 0.534 (.003) | -40 | 0.000 | 0.646 (.039) | 0.641 (.017) | -1 | 0.859 |
|  | **Polg2** | 0.080 (.001) | 0.116 (.005) | 45 | 0.005 | 0.090 (.007) | 0.148 (.015) | 64 | 0.004 | 0.129 (.002) | 0.112 (.031) | -13 | 0.452 |
|  | **Tfam** | 0.106 (.018) | 0.108 (.004) | 2 | 0.847 | 0.158 (.011) | 0.158 (.003) | 0 | 0.961 | 0.135 (.002) | 0.128 (.009) | -5 | 0.345 |
|  | **Fis1** | 0.058 (.001) | 0.058 (.006) | 0 | 1.000 | 0.063 (.007) | 0.058 (.004) | -7 | 0.387 | 0.063 (.013) | 0.050 (.002) | -20 | 0.231 |
|  | **Opa1** | 0.073 (.005) | 0.085 (.011) | 16 | 0.169 | 0.063 (.011) | 0.081 (.009) | 28 | 0.095 | 0.064 (.001) | 0.075 (.006) | 18 | 0.075 |
|  |  |  |  |  |  |  |  |  |  |  |  |  |  |
| **Kidney** | **Polg** | 0.633 (.003) | 0.517 (.021) | -18 | 0.010 | 0.562 (.041) | 0.552 (.042) | -2 | 0.797 | 0.583 (.031) | 0.567 (.011) | -3 | 0.448 |
|  | **Polg2** | 0.114 (.012) | 0.104 (.004) | -9 | 0.245 | 0.121 (.009) | 0.179 (.013) | 48 | 0.003 | 0.090 (.008) | 0.132 (.005) | 47 | 0.001 |
|  | **Tfam** | 0.148 (.002) | 0.125 (.001) | -15 | 0.000 | 0.166 (.005) | 0.150 (.005) | -10 | 0.012 | 0.205 (.011) | 0.210 (.006) | 2 | 0.508 |
|  | **Fis1** | 0.051 (.005) | 0.059 (.004) | 14 | 0.097 | 0.050 (.002) | 0.055 (.003) | 10 | 0.096 | 0.046 (.002) | 0.045 (.003) | -2 | 0.639 |
|  | **Opa1** | 0.070 (.010) | 0.079 (.004) | 13 | 0.210 | 0.072 (.011) | 0.063 (.002) | -13 | 0.216 | 0.080 (.000) | 0.089 (.006) | 11 | 0.130 |
|  |  |  |  |  |  |  |  |  |  |  |  |  |  |
| **Liver** | **Polg** | 0.500 (.006) | 0.450 (.006) | -10 | 0.000 | 0.258 (.009) | 0.267 (.007) | 3 | 0.275 | 0.202 (.029) | 0.460 (.004) | 128 | 0.004 |
|  | **Polg2** | 0.095 (.020) | 0.122 (.002) | 29 | 0.138 | 0.084 (.018) | 0.077 (.021) | -8 | 0.703 | 0.117 (.006) | 0.141 (.002) | 20 | 0.002 |
|  | **Tfam** | 0.138 (.002) | 0.152 (.002) | 10 | 0.001 | 0.146 (.007) | 0.177 (.007) | 21 | 0.006 | 0.170 (.013) | 0.175 (.010) | 3 | 0.649 |
|  | **Fis1** | 0.047 (.001) | 0.062 (.012) | 30 | 0.109 | 0.056 (.006) | 0.055 (.007) | -1 | 0.905 | 0.054 (.005) | 0.055 (.009) | 1 | 0.915 |
|  | **Opa1** | 0.061 (.002) | 0.068 (.004) | 10 | 0.073 | 0.078 (.006) | 0.088 (.001) | 12 | 0.050 | 0.088 (.004) | 0.092 (.005) | 5 | 0.318 |
