# Supplementary material for: Multi-Tissue DNA Methylation Remodeling at Mitochondrial Quality Control Genes According to Diet in Rat Aging Models
Source: Nutrients. 2020 Feb 12;12(2):460. doi: 10.3390/nu12020460 (PMC7071227; doi:10.3390/nu12020460)
Supplement: Supplementary file 1 [file nutrients-12-00460-s001.zip › nutrients-721651-supplementary/Table S4.docx]

**Table S4.** DNA methylation values of the CpG sites of each gene according to age and tissues in rats fed standard or low-calorie diet. SD: standard deviation.

| **Blood** | **27 weeks** | | | | | | | | **36 weeks** | | | | | | | | **96 weeks** | | | | | | | |
| --- | --- | --- | --- | --- | --- | --- | --- | --- | --- | --- | --- | --- | --- | --- | --- | --- | --- | --- | --- | --- | --- | --- | --- | --- |
|  | **Standard diet** | | | | **Low-calorie diet** | | | | **Standard diet** | | | | **Low-calorie diet** | | | | **Standard diet** | | | | **Low-calorie diet** | | | |
|  | **Mean (SD)** | | | | **Mean (SD)** | | | | **Mean (SD)** | | | | **Mean (SD)** | | | | **Mean (SD)** | | | | **Mean (SD)** | | | |
| POLG_CpG_1 | 0.563 | ( | 0.006 | ) | 0.510 | ( | 0.010 | ) | 0.627 | ( | 0.006 | ) | 0.520 | ( | 0.082 | ) | 0.663 | ( | 0.029 | ) | 0.663 | ( | 0.006 | ) |
| POLG_CpG_2 | 0.447 | ( | 0.006 | ) | 0.430 | ( | 0.030 | ) | 0.447 | ( | 0.006 | ) | 0.430 | ( | 0.062 | ) | 0.430 | ( | 0.017 | ) | 0.583 | ( | 0.091 | ) |
| POLG_CpG_6.7 | 0.327 | ( | 0.006 | ) | 0.207 | ( | 0.006 | ) | 0.317 | ( | 0.006 | ) | 0.217 | ( | 0.006 | ) | 0.327 | ( | 0.006 | ) | 0.227 | ( | 0.006 | ) |
| POLG_CpG_9 | 0.293 | ( | 0.006 | ) | 0.307 | ( | 0.006 | ) | 0.297 | ( | 0.006 | ) | 0.300 | ( | 0.000 | ) | 0.307 | ( | 0.006 | ) | 0.303 | ( | 0.006 | ) |
| POLG_CpG_11.12 | 0.397 | ( | 0.006 | ) | 0.397 | ( | 0.006 | ) | 0.397 | ( | 0.006 | ) | 0.993 | ( | 0.006 | ) | 0.407 | ( | 0.006 | ) | 0.980 | ( | 0.035 | ) |
| POLG_CpG_13 | 0.640 | ( | 0.010 | ) | 0.870 | ( | 0.026 | ) | 0.627 | ( | 0.006 | ) | 0.897 | ( | 0.038 | ) | 0.650 | ( | 0.017 | ) | 0.887 | ( | 0.012 | ) |
| POLG_CpG_14 | 0.627 | ( | 0.006 | ) | 0.903 | ( | 0.006 | ) | 0.617 | ( | 0.006 | ) | 0.903 | ( | 0.057 | ) | 0.600 | ( | 0.017 | ) | 0.990 | ( | 0.010 | ) |
| POLG_CpG_15 | 0.397 | ( | 0.006 | ) | 0.980 | ( | 0.010 | ) | 0.427 | ( | 0.006 | ) | 0.973 | ( | 0.021 | ) | 0.463 | ( | 0.029 | ) | 0.947 | ( | 0.032 | ) |
| POLG2_CpG_1 | 0.043 | ( | 0.021 | ) | 0.550 | ( | 0.217 | ) | 0.007 | ( | 0.006 | ) | 0.653 | ( | 0.222 | ) | 0.037 | ( | 0.006 | ) | 0.780 | ( | 0.072 | ) |
| POLG2_CpG_2 | 0.070 | ( | 0.026 | ) | 0.127 | ( | 0.006 | ) | 0.017 | ( | 0.012 | ) | 0.000 | ( | 0.000 | ) | 0.030 | ( | 0.000 | ) | 0.000 | ( | 0.000 | ) |
| POLG2_CpG_3.4 | 0.220 | ( | 0.044 | ) | 0.000 | ( | 0.000 | ) | 0.083 | ( | 0.006 | ) | 0.210 | ( | 0.182 | ) | 0.110 | ( | 0.052 | ) | 0.013 | ( | 0.006 | ) |
| POLG2_CpG_5 | 0.267 | ( | 0.006 | ) | 0.057 | ( | 0.006 | ) | 0.283 | ( | 0.021 | ) | 0.070 | ( | 0.000 | ) | 0.247 | ( | 0.006 | ) | 0.030 | ( | 0.000 | ) |
| POLG2_CpG_6.7.8 | 0.200 | ( | 0.061 | ) | 0.260 | ( | 0.000 | ) | 0.247 | ( | 0.006 | ) | 0.243 | ( | 0.012 | ) | 0.237 | ( | 0.023 | ) | 0.293 | ( | 0.012 | ) |
| POLG2_CpG_9.10 | 0.117 | ( | 0.032 | ) | 0.237 | ( | 0.047 | ) | 0.107 | ( | 0.006 | ) | 0.183 | ( | 0.015 | ) | 0.110 | ( | 0.000 | ) | 0.190 | ( | 0.036 | ) |
| POLG2_CpG_11 | 0.100 | ( | 0.010 | ) | 0.100 | ( | 0.010 | ) | 0.053 | ( | 0.006 | ) | 0.100 | ( | 0.030 | ) | 0.067 | ( | 0.012 | ) | 0.093 | ( | 0.006 | ) |
| POLG2_CpG_12 | 0.150 | ( | 0.026 | ) | 0.067 | ( | 0.006 | ) | 0.137 | ( | 0.006 | ) | 0.000 | ( | 0.000 | ) | 0.093 | ( | 0.040 | ) | 0.040 | ( | 0.020 | ) |
| POLG2_CpG_13 | 0.117 | ( | 0.015 | ) | 0.427 | ( | 0.025 | ) | 0.067 | ( | 0.006 | ) | 0.000 | ( | 0.000 | ) | 0.100 | ( | 0.000 | ) | 0.190 | ( | 0.010 | ) |
| POLG2_CpG_14 | 0.037 | ( | 0.015 | ) | 0.507 | ( | 0.006 | ) | 0.047 | ( | 0.006 | ) | 0.203 | ( | 0.006 | ) | 0.033 | ( | 0.012 | ) | 0.107 | ( | 0.012 | ) |
| POLG2_CpG_15 | 0.050 | ( | 0.026 | ) | 0.010 | ( | 0.000 | ) | 0.080 | ( | 0.026 | ) | 0.010 | ( | 0.000 | ) | 0.037 | ( | 0.006 | ) | 0.010 | ( | 0.000 | ) |
| POLG2_CpG_16 | 0.063 | ( | 0.006 | ) | 0.050 | ( | 0.000 | ) | 0.097 | ( | 0.006 | ) | 0.070 | ( | 0.026 | ) | 0.037 | ( | 0.006 | ) | 0.053 | ( | 0.006 | ) |
| POLG2_CpG_17.18 | 0.250 | ( | 0.026 | ) | 0.087 | ( | 0.006 | ) | 0.277 | ( | 0.006 | ) | 0.267 | ( | 0.006 | ) | 0.227 | ( | 0.012 | ) | 0.207 | ( | 0.006 | ) |
| POLG2_CpG_19 | 0.027 | ( | 0.006 | ) | 0.220 | ( | 0.098 | ) | 0.010 | ( | 0.000 | ) | 0.287 | ( | 0.095 | ) | 0.000 | ( | 0.000 | ) | 0.283 | ( | 0.081 | ) |
| POLG2_CpG_20.21 | 0.260 | ( | 0.010 | ) | 0.173 | ( | 0.006 | ) | 0.277 | ( | 0.006 | ) | 0.000 | ( | 0.000 | ) | 0.227 | ( | 0.012 | ) | 0.207 | ( | 0.006 | ) |
| POLG2_CpG_22 | 0.033 | ( | 0.015 | ) | 0.220 | ( | 0.098 | ) | 0.067 | ( | 0.006 | ) | 0.287 | ( | 0.095 | ) | 0.030 | ( | 0.000 | ) | 0.283 | ( | 0.081 | ) |
| POLG2_CpG_23 | 0.053 | ( | 0.006 | ) | 0.093 | ( | 0.006 | ) | 0.280 | ( | 0.052 | ) | 0.103 | ( | 0.006 | ) | 0.030 | ( | 0.000 | ) | 0.010 | ( | 0.010 | ) |
| POLG2_CpG_24 | 0.030 | ( | 0.010 | ) | 0.063 | ( | 0.050 | ) | 0.067 | ( | 0.006 | ) | 0.150 | ( | 0.010 | ) | 0.027 | ( | 0.006 | ) | 0.130 | ( | 0.122 | ) |
| POLG2_CpG_25.26.27 | 0.047 | ( | 0.006 | ) | 0.093 | ( | 0.006 | ) | 0.017 | ( | 0.006 | ) | 0.100 | ( | 0.010 | ) | 0.020 | ( | 0.000 | ) | 0.007 | ( | 0.006 | ) |
| POLG2_CpG_28.29.30 | 0.057 | ( | 0.015 | ) | 0.030 | ( | 0.000 | ) | 0.030 | ( | 0.010 | ) | 0.020 | ( | 0.000 | ) | 0.027 | ( | 0.006 | ) | 0.010 | ( | 0.000 | ) |
| POLG2_CpG_31.32 | 0.110 | ( | 0.056 | ) | 0.000 | ( | 0.000 | ) | 0.047 | ( | 0.012 | ) | 0.020 | ( | 0.010 | ) | 0.047 | ( | 0.006 | ) | 0.010 | ( | 0.000 | ) |
| POLG2_CpG_33.34 | 0.097 | ( | 0.035 | ) | 0.040 | ( | 0.040 | ) | 0.173 | ( | 0.015 | ) | 0.033 | ( | 0.025 | ) | 0.113 | ( | 0.064 | ) | 0.030 | ( | 0.000 | ) |
| POLG2_CpG_35.36 | 0.120 | ( | 0.010 | ) | 0.170 | ( | 0.050 | ) | 0.103 | ( | 0.006 | ) | 0.197 | ( | 0.012 | ) | 0.110 | ( | 0.000 | ) | 0.087 | ( | 0.032 | ) |
| POLG2_CpG_37.38.39.40 | 0.100 | ( | 0.010 | ) | 0.100 | ( | 0.010 | ) | 0.090 | ( | 0.010 | ) | 0.100 | ( | 0.030 | ) | 0.093 | ( | 0.012 | ) | 0.093 | ( | 0.006 | ) |
| POLG2_CpG_41 | 0.047 | ( | 0.006 | ) | 0.020 | ( | 0.000 | ) | 0.023 | ( | 0.006 | ) | 0.020 | ( | 0.000 | ) | 0.030 | ( | 0.000 | ) | 0.020 | ( | 0.000 | ) |
| TFAM_CpG_1 | 0.047 | ( | 0.006 | ) | 0.040 | ( | 0.026 | ) | 0.067 | ( | 0.006 | ) | 0.077 | ( | 0.015 | ) | 0.077 | ( | 0.006 | ) | 0.040 | ( | 0.010 | ) |
| TFAM_CpG_2.3 | 0.040 | ( | 0.010 | ) | 0.047 | ( | 0.006 | ) | 0.060 | ( | 0.010 | ) | 0.057 | ( | 0.006 | ) | 0.067 | ( | 0.032 | ) | 0.063 | ( | 0.031 | ) |
| TFAM_CpG_4 | 0.043 | ( | 0.006 | ) | 0.050 | ( | 0.000 | ) | 0.063 | ( | 0.006 | ) | 0.040 | ( | 0.000 | ) | 0.073 | ( | 0.006 | ) | 0.073 | ( | 0.006 | ) |
| TFAM_CpG_5 | 0.917 | ( | 0.085 | ) | 0.887 | ( | 0.032 | ) | 0.913 | ( | 0.150 | ) | 0.880 | ( | 0.010 | ) | 0.537 | ( | 0.084 | ) | 0.540 | ( | 0.087 | ) |
| TFAM_CpG_6 | 0.043 | ( | 0.006 | ) | 0.043 | ( | 0.006 | ) | 0.063 | ( | 0.006 | ) | 0.070 | ( | 0.010 | ) | 0.073 | ( | 0.006 | ) | 0.077 | ( | 0.006 | ) |
| TFAM_CpG_7 | 0.033 | ( | 0.015 | ) | 0.037 | ( | 0.015 | ) | 0.053 | ( | 0.015 | ) | 0.050 | ( | 0.000 | ) | 0.057 | ( | 0.015 | ) | 0.060 | ( | 0.010 | ) |
| TFAM_CpG_8 | 0.243 | ( | 0.015 | ) | 0.247 | ( | 0.031 | ) | 0.263 | ( | 0.015 | ) | 0.297 | ( | 0.006 | ) | 0.150 | ( | 0.061 | ) | 0.180 | ( | 0.010 | ) |
| TFAM_CpG_12 | 0.007 | ( | 0.006 | ) | 0.007 | ( | 0.006 | ) | 0.017 | ( | 0.015 | ) | 0.010 | ( | 0.000 | ) | 0.043 | ( | 0.006 | ) | 0.047 | ( | 0.006 | ) |
| FIS_1_CpG_1 | 0.000 | ( | 0.000 | ) | 0.023 | ( | 0.021 | ) | 0.020 | ( | 0.000 | ) | 0.053 | ( | 0.015 | ) | 0.010 | ( | 0.000 | ) | 0.017 | ( | 0.015 | ) |
| FIS_1_CpG_3 | 0.000 | ( | 0.000 | ) | 0.017 | ( | 0.015 | ) | 0.000 | ( | 0.000 | ) | 0.030 | ( | 0.026 | ) | 0.000 | ( | 0.000 | ) | 0.007 | ( | 0.006 | ) |
| FIS_1_CpG_4 | 0.050 | ( | 0.000 | ) | 0.070 | ( | 0.017 | ) | 0.040 | ( | 0.000 | ) | 0.077 | ( | 0.015 | ) | 0.043 | ( | 0.006 | ) | 0.057 | ( | 0.012 | ) |
| FIS_1_CpG_5 | 0.000 | ( | 0.000 | ) | 0.000 | ( | 0.000 | ) | 0.017 | ( | 0.006 | ) | 0.000 | ( | 0.000 | ) | 0.017 | ( | 0.012 | ) | 0.023 | ( | 0.006 | ) |
| FIS1_1_CpG_6 | 0.037 | ( | 0.006 | ) | 0.063 | ( | 0.006 | ) | 0.050 | ( | 0.000 | ) | 0.060 | ( | 0.020 | ) | 0.037 | ( | 0.006 | ) | 0.037 | ( | 0.006 | ) |
| FIS1_1_CpG_7.8 | 0.040 | ( | 0.000 | ) | 0.040 | ( | 0.000 | ) | 0.050 | ( | 0.000 | ) | 0.043 | ( | 0.015 | ) | 0.050 | ( | 0.000 | ) | 0.030 | ( | 0.000 | ) |
| FIS1_1_CpG_9 | 0.103 | ( | 0.006 | ) | 0.100 | ( | 0.061 | ) | 0.300 | ( | 0.052 | ) | 0.040 | ( | 0.017 | ) | 0.290 | ( | 0.139 | ) | 0.057 | ( | 0.006 | ) |
| FIS1_1_CpG_11 | 0.027 | ( | 0.006 | ) | 0.070 | ( | 0.010 | ) | 0.030 | ( | 0.000 | ) | 0.030 | ( | 0.000 | ) | 0.040 | ( | 0.000 | ) | 0.037 | ( | 0.035 | ) |
| FIS1_2_CpG_6 | 0.033 | ( | 0.021 | ) | 0.027 | ( | 0.006 | ) | 0.027 | ( | 0.006 | ) | 0.030 | ( | 0.017 | ) | 0.017 | ( | 0.006 | ) | 0.017 | ( | 0.006 | ) |
| FIS1_2_CpG_7 | 0.060 | ( | 0.044 | ) | 0.023 | ( | 0.006 | ) | 0.043 | ( | 0.012 | ) | 0.020 | ( | 0.000 | ) | 0.017 | ( | 0.015 | ) | 0.007 | ( | 0.006 | ) |
| FIS1_2_CpG_8 | 0.010 | ( | 0.010 | ) | 0.030 | ( | 0.000 | ) | 0.010 | ( | 0.000 | ) | 0.010 | ( | 0.000 | ) | 0.000 | ( | 0.000 | ) | 0.020 | ( | 0.020 | ) |
| FIS1_2_CpG_10 | 0.103 | ( | 0.006 | ) | 0.110 | ( | 0.010 | ) | 0.090 | ( | 0.000 | ) | 0.093 | ( | 0.006 | ) | 0.097 | ( | 0.006 | ) | 0.083 | ( | 0.015 | ) |
| FIS1_2_CpG_11.12.13 | 0.033 | ( | 0.006 | ) | 0.037 | ( | 0.006 | ) | 0.030 | ( | 0.000 | ) | 0.023 | ( | 0.006 | ) | 0.027 | ( | 0.006 | ) | 0.030 | ( | 0.010 | ) |
| FIS1_2_CpG_14.15 | 0.023 | ( | 0.006 | ) | 0.013 | ( | 0.006 | ) | 0.000 | ( | 0.000 | ) | 0.020 | ( | 0.010 | ) | 0.017 | ( | 0.015 | ) | 0.020 | ( | 0.000 | ) |
| FIS1_2_CpG_16.17.18 | 0.087 | ( | 0.032 | ) | 0.147 | ( | 0.015 | ) | 0.117 | ( | 0.012 | ) | 0.140 | ( | 0.000 | ) | 0.100 | ( | 0.026 | ) | 0.120 | ( | 0.020 | ) |
| OPA1_1_CpG_1 | 0.127 | ( | 0.067 | ) | 0.057 | ( | 0.006 | ) | 0.047 | ( | 0.040 | ) | 0.083 | ( | 0.006 | ) | 0.047 | ( | 0.006 | ) | 0.053 | ( | 0.021 | ) |
| OPA1_1_CpG_2 | 0.037 | ( | 0.006 | ) | 0.047 | ( | 0.006 | ) | 0.043 | ( | 0.006 | ) | 0.050 | ( | 0.010 | ) | 0.070 | ( | 0.010 | ) | 0.043 | ( | 0.006 | ) |
| OPA1_1_CpG_3 | 0.040 | ( | 0.026 | ) | 0.047 | ( | 0.006 | ) | 0.047 | ( | 0.006 | ) | 0.010 | ( | 0.000 | ) | 0.033 | ( | 0.006 | ) | 0.027 | ( | 0.006 | ) |
| OPA1_1_CpG_4 | 0.037 | ( | 0.021 | ) | 0.027 | ( | 0.006 | ) | 0.023 | ( | 0.012 | ) | 0.023 | ( | 0.012 | ) | 0.020 | ( | 0.010 | ) | 0.027 | ( | 0.006 | ) |
| OPA1_1_CpG_5.6 | 0.050 | ( | 0.010 | ) | 0.067 | ( | 0.006 | ) | 0.093 | ( | 0.006 | ) | 0.110 | ( | 0.046 | ) | 0.137 | ( | 0.067 | ) | 0.093 | ( | 0.021 | ) |
| OPA1_1_CpG_7 | 0.060 | ( | 0.010 | ) | 0.067 | ( | 0.006 | ) | 0.057 | ( | 0.006 | ) | 0.037 | ( | 0.006 | ) | 0.047 | ( | 0.006 | ) | 0.047 | ( | 0.006 | ) |
| OPA1_1_CpG_8 | 0.013 | ( | 0.006 | ) | 0.027 | ( | 0.006 | ) | 0.017 | ( | 0.006 | ) | 0.017 | ( | 0.006 | ) | 0.023 | ( | 0.006 | ) | 0.020 | ( | 0.010 | ) |
| OPA1_2_CpG_1 | 0.073 | ( | 0.006 | ) | 0.137 | ( | 0.006 | ) | 0.047 | ( | 0.006 | ) | 0.047 | ( | 0.006 | ) | 0.017 | ( | 0.006 | ) | 0.097 | ( | 0.006 | ) |
| OPA1_2_CpG_3 | 0.010 | ( | 0.000 | ) | 0.010 | ( | 0.000 | ) | 0.000 | ( | 0.000 | ) | 0.010 | ( | 0.000 | ) | 0.020 | ( | 0.010 | ) | 0.010 | ( | 0.000 | ) |
| OPA1_2_CpG_4 | 0.037 | ( | 0.006 | ) | 0.063 | ( | 0.006 | ) | 0.073 | ( | 0.006 | ) | 0.070 | ( | 0.044 | ) | 0.017 | ( | 0.006 | ) | 0.057 | ( | 0.015 | ) |
| OPA1_2_CpG_5 | 0.027 | ( | 0.006 | ) | 0.013 | ( | 0.006 | ) | 0.070 | ( | 0.000 | ) | 0.120 | ( | 0.010 | ) | 0.017 | ( | 0.006 | ) | 0.057 | ( | 0.015 | ) |
| OPA1_2_CpG_6 | 0.577 | ( | 0.045 | ) | 0.013 | ( | 0.006 | ) | 0.430 | ( | 0.020 | ) | 0.567 | ( | 0.032 | ) | 0.447 | ( | 0.006 | ) | 0.507 | ( | 0.162 | ) |
| OPA1_2_CpG_7 | 0.010 | ( | 0.000 | ) | 0.393 | ( | 0.006 | ) | 0.030 | ( | 0.000 | ) | 0.010 | ( | 0.000 | ) | 0.017 | ( | 0.006 | ) | 0.010 | ( | 0.000 | ) |
| OPA1_2_CpG_8 | 0.000 | ( | 0.000 | ) | 0.093 | ( | 0.006 | ) | 0.040 | ( | 0.000 | ) | 0.010 | ( | 0.000 | ) | 0.037 | ( | 0.006 | ) | 0.040 | ( | 0.000 | ) |
| OPA1_2_CpG_9 | 0.020 | ( | 0.000 | ) | 0.043 | ( | 0.006 | ) | 0.000 | ( | 0.000 | ) | 0.010 | ( | 0.000 | ) | 0.130 | ( | 0.000 | ) | 0.133 | ( | 0.006 | ) |
| OPA1_2_CpG_10.11 | 0.033 | ( | 0.006 | ) | 0.010 | ( | 0.000 | ) | 0.367 | ( | 0.025 | ) | 0.407 | ( | 0.012 | ) | 0.017 | ( | 0.006 | ) | 0.020 | ( | 0.000 | ) |
| OPA1_2_CpG_12 | 0.580 | ( | 0.044 | ) | 0.103 | ( | 0.006 | ) | 0.417 | ( | 0.021 | ) | 0.540 | ( | 0.036 | ) | 0.450 | ( | 0.010 | ) | 0.333 | ( | 0.015 | ) |
| OPA1_2_CpG_13 | 0.010 | ( | 0.000 | ) | 0.393 | ( | 0.006 | ) | 0.000 | ( | 0.000 | ) | 0.010 | ( | 0.000 | ) | 0.010 | ( | 0.010 | ) | 0.027 | ( | 0.012 | ) |
| OPA1_2_CpG_14.15 | 0.020 | ( | 0.000 | ) | 0.010 | ( | 0.000 | ) | 0.043 | ( | 0.006 | ) | 0.023 | ( | 0.006 | ) | 0.027 | ( | 0.006 | ) | 0.033 | ( | 0.012 | ) |
| OPA1_2_CpG_16 | 0.023 | ( | 0.006 | ) | 0.043 | ( | 0.006 | ) | 0.057 | ( | 0.006 | ) | 0.030 | ( | 0.010 | ) | 0.047 | ( | 0.006 | ) | 0.107 | ( | 0.015 | ) |
| OPA1_3_CpG_1 | 0.073 | ( | 0.006 | ) | 0.037 | ( | 0.006 | ) | 0.000 | ( | 0.000 | ) | 0.020 | ( | 0.010 | ) | 0.010 | ( | 0.000 | ) | 0.073 | ( | 0.006 | ) |
| OPA1_3_CpG_2.3 | 0.057 | ( | 0.006 | ) | 0.043 | ( | 0.015 | ) | 0.080 | ( | 0.017 | ) | 0.043 | ( | 0.042 | ) | 0.010 | ( | 0.000 | ) | 0.043 | ( | 0.015 | ) |
| OPA1_3_CpG_4 | 0.073 | ( | 0.015 | ) | 0.060 | ( | 0.010 | ) | 0.000 | ( | 0.000 | ) | 0.140 | ( | 0.017 | ) | 0.050 | ( | 0.010 | ) | 0.100 | ( | 0.010 | ) |
| OPA1_3_CpG_5 | 0.043 | ( | 0.006 | ) | 0.050 | ( | 0.010 | ) | 0.067 | ( | 0.006 | ) | 0.050 | ( | 0.010 | ) | 0.030 | ( | 0.010 | ) | 0.037 | ( | 0.006 | ) |
| OPA1_3_CpG_6.7 | 0.057 | ( | 0.012 | ) | 0.050 | ( | 0.010 | ) | 0.020 | ( | 0.017 | ) | 0.027 | ( | 0.015 | ) | 0.030 | ( | 0.010 | ) | 0.027 | ( | 0.012 | ) |
| OPA1_3_CpG_8.9 | 0.097 | ( | 0.012 | ) | 0.137 | ( | 0.038 | ) | 0.093 | ( | 0.023 | ) | 0.107 | ( | 0.074 | ) | 0.180 | ( | 0.010 | ) | 0.107 | ( | 0.025 | ) |
| OPA1_3_CpG_11.12 | 0.010 | ( | 0.000 | ) | 0.023 | ( | 0.015 | ) | 0.030 | ( | 0.017 | ) | 0.013 | ( | 0.012 | ) | 0.017 | ( | 0.006 | ) | 0.037 | ( | 0.006 | ) |
| OPA1_3_CpG_13.14.15.16 | 0.037 | ( | 0.006 | ) | 0.037 | ( | 0.006 | ) | 0.010 | ( | 0.000 | ) | 0.023 | ( | 0.012 | ) | 0.020 | ( | 0.010 | ) | 0.033 | ( | 0.015 | ) |
| OPA1_3_CpG_17 | 0.043 | ( | 0.006 | ) | 0.050 | ( | 0.010 | ) | 0.010 | ( | 0.000 | ) | 0.020 | ( | 0.010 | ) | 0.020 | ( | 0.010 | ) | 0.023 | ( | 0.015 | ) |
| OPA1_3_CpG_19 | 0.040 | ( | 0.000 | ) | 0.047 | ( | 0.006 | ) | 0.030 | ( | 0.000 | ) | 0.040 | ( | 0.010 | ) | 0.040 | ( | 0.010 | ) | 0.033 | ( | 0.006 | ) |
| OPA1_3_CpG_20 | 0.043 | ( | 0.006 | ) | 0.033 | ( | 0.015 | ) | 0.037 | ( | 0.012 | ) | 0.020 | ( | 0.010 | ) | 0.010 | ( | 0.000 | ) | 0.030 | ( | 0.010 | ) |
|  |  |  |  |  |  |  |  |  |  |  |  |  |  |  |  |  |  |  |  |  |  |  |  |  |
| **Heart** | **27 weeks** | | | | | | | | **36 weeks** | | | | | | | | **96 weeks** | | | | | | | |
|  | **Standard diet** | | | | **Low-calorie diet** | | | | **Standard diet** | | | | **Low-calorie diet** | | | | **Standard diet** | | | | **Low-calorie diet** | | | |
|  | **Mean (SD)** | | | | **Mean (SD)** | | | | **Mean (SD)** | | | | **Mean (SD)** | | | | **Mean (SD)** | | | | **Mean (SD)** | | | |
| POLG_CpG_1 | 0.697 | ( | 0.127 | ) | 0.627 | ( | 0.127 | ) | 0.650 | ( | 0.017 | ) | 0.587 | ( | 0.006 | ) | 0.757 | ( | 0.023 | ) | 0.743 | ( | 0.046 | ) |
| POLG_CpG_2 | 0.360 | ( | 0.052 | ) | 0.733 | ( | 0.006 | ) | 0.860 | ( | 0.017 | ) | 0.213 | ( | 0.015 | ) | 0.823 | ( | 0.081 | ) | 0.823 | ( | 0.042 | ) |
| POLG_CpG_6.7 | 0.313 | ( | 0.021 | ) | 0.323 | ( | 0.012 | ) | 0.960 | ( | 0.000 | ) | 0.960 | ( | 0.000 | ) | 0.493 | ( | 0.040 | ) | 0.480 | ( | 0.017 | ) |
| POLG_CpG_9 | 0.273 | ( | 0.040 | ) | 0.310 | ( | 0.010 | ) | 0.993 | ( | 0.006 | ) | 0.633 | ( | 0.006 | ) | 0.517 | ( | 0.150 | ) | 0.570 | ( | 0.069 | ) |
| POLG_CpG_11.12 | 0.380 | ( | 0.035 | ) | 0.313 | ( | 0.006 | ) | 0.860 | ( | 0.035 | ) | 0.293 | ( | 0.012 | ) | 0.543 | ( | 0.075 | ) | 0.530 | ( | 0.052 | ) |
| POLG_CpG_13 | 0.653 | ( | 0.058 | ) | 0.433 | ( | 0.006 | ) | 0.963 | ( | 0.012 | ) | 0.353 | ( | 0.012 | ) | 0.807 | ( | 0.064 | ) | 0.783 | ( | 0.023 | ) |
| POLG_CpG_14 | 0.610 | ( | 0.035 | ) | 0.663 | ( | 0.006 | ) | 0.957 | ( | 0.049 | ) | 0.630 | ( | 0.010 | ) | 0.767 | ( | 0.064 | ) | 0.753 | ( | 0.040 | ) |
| POLG_CpG_15 | 0.410 | ( | 0.104 | ) | 0.623 | ( | 0.006 | ) | 0.883 | ( | 0.006 | ) | 0.603 | ( | 0.012 | ) | 0.457 | ( | 0.029 | ) | 0.443 | ( | 0.006 | ) |
| POLG2_CpG_1 | 0.053 | ( | 0.006 | ) | 0.453 | ( | 0.006 | ) | 0.010 | ( | 0.000 | ) | 0.557 | ( | 0.025 | ) | 0.027 | ( | 0.006 | ) | 0.000 | ( | 0.000 | ) |
| POLG2_CpG_2 | 0.027 | ( | 0.006 | ) | 0.093 | ( | 0.006 | ) | 0.010 | ( | 0.000 | ) | 0.057 | ( | 0.006 | ) | 0.100 | ( | 0.000 | ) | 0.093 | ( | 0.006 | ) |
| POLG2_CpG_3.4 | 0.093 | ( | 0.023 | ) | 0.013 | ( | 0.006 | ) | 0.100 | ( | 0.000 | ) | 0.103 | ( | 0.064 | ) | 0.057 | ( | 0.012 | ) | 0.053 | ( | 0.006 | ) |
| POLG2_CpG_5 | 0.303 | ( | 0.006 | ) | 0.103 | ( | 0.006 | ) | 0.333 | ( | 0.042 | ) | 0.063 | ( | 0.015 | ) | 0.453 | ( | 0.015 | ) | 0.443 | ( | 0.031 | ) |
| POLG2_CpG_6.7.8 | 0.273 | ( | 0.006 | ) | 0.333 | ( | 0.006 | ) | 0.210 | ( | 0.026 | ) | 0.220 | ( | 0.010 | ) | 0.230 | ( | 0.000 | ) | 0.217 | ( | 0.023 | ) |
| POLG2_CpG_9.10 | 0.107 | ( | 0.006 | ) | 0.313 | ( | 0.006 | ) | 0.100 | ( | 0.010 | ) | 0.250 | ( | 0.030 | ) | 0.107 | ( | 0.006 | ) | 0.093 | ( | 0.029 | ) |
| POLG2_CpG_11 | 0.103 | ( | 0.006 | ) | 0.093 | ( | 0.006 | ) | 0.073 | ( | 0.012 | ) | 0.090 | ( | 0.010 | ) | 0.083 | ( | 0.012 | ) | 0.073 | ( | 0.038 | ) |
| POLG2_CpG_12 | 0.010 | ( | 0.000 | ) | 0.023 | ( | 0.006 | ) | 0.027 | ( | 0.025 | ) | 0.067 | ( | 0.055 | ) | 0.130 | ( | 0.000 | ) | 0.117 | ( | 0.023 | ) |
| POLG2_CpG_13 | 0.057 | ( | 0.046 | ) | 0.283 | ( | 0.006 | ) | 0.177 | ( | 0.143 | ) | 0.000 | ( | 0.000 | ) | 0.293 | ( | 0.006 | ) | 0.217 | ( | 0.136 | ) |
| POLG2_CpG_14 | 0.047 | ( | 0.006 | ) | 0.010 | ( | 0.000 | ) | 0.073 | ( | 0.006 | ) | 0.387 | ( | 0.015 | ) | 0.000 | ( | 0.000 | ) | 0.030 | ( | 0.000 | ) |
| POLG2_CpG_15 | 0.010 | ( | 0.000 | ) | 0.083 | ( | 0.006 | ) | 0.000 | ( | 0.000 | ) | 0.147 | ( | 0.006 | ) | 0.010 | ( | 0.000 | ) | 0.020 | ( | 0.017 | ) |
| POLG2_CpG_16 | 0.010 | ( | 0.010 | ) | 0.010 | ( | 0.000 | ) | 0.000 | ( | 0.000 | ) | 0.010 | ( | 0.000 | ) | 0.000 | ( | 0.000 | ) | 0.037 | ( | 0.006 | ) |
| POLG2_CpG_17.18 | 0.183 | ( | 0.029 | ) | 0.093 | ( | 0.006 | ) | 0.207 | ( | 0.086 | ) | 0.210 | ( | 0.075 | ) | 0.313 | ( | 0.012 | ) | 0.250 | ( | 0.113 | ) |
| POLG2_CpG_19 | 0.010 | ( | 0.000 | ) | 0.123 | ( | 0.006 | ) | 0.010 | ( | 0.000 | ) | 0.230 | ( | 0.096 | ) | 0.023 | ( | 0.006 | ) | 0.020 | ( | 0.010 | ) |
| POLG2_CpG_20.21 | 0.183 | ( | 0.029 | ) | 0.193 | ( | 0.012 | ) | 0.207 | ( | 0.086 | ) | 0.143 | ( | 0.012 | ) | 0.313 | ( | 0.012 | ) | 0.253 | ( | 0.115 | ) |
| POLG2_CpG_22 | 0.000 | ( | 0.000 | ) | 0.123 | ( | 0.006 | ) | 0.023 | ( | 0.006 | ) | 0.230 | ( | 0.096 | ) | 0.110 | ( | 0.010 | ) | 0.097 | ( | 0.025 | ) |
| POLG2_CpG_23 | 0.023 | ( | 0.012 | ) | 0.020 | ( | 0.000 | ) | 0.110 | ( | 0.036 | ) | 0.123 | ( | 0.025 | ) | 0.193 | ( | 0.006 | ) | 0.130 | ( | 0.104 | ) |
| POLG2_CpG_24 | 0.000 | ( | 0.000 | ) | 0.053 | ( | 0.006 | ) | 0.010 | ( | 0.000 | ) | 0.257 | ( | 0.006 | ) | 0.110 | ( | 0.010 | ) | 0.097 | ( | 0.025 | ) |
| POLG2_CpG_25.26.27 | 0.067 | ( | 0.006 | ) | 0.053 | ( | 0.006 | ) | 0.100 | ( | 0.000 | ) | 0.093 | ( | 0.006 | ) | 0.120 | ( | 0.017 | ) | 0.090 | ( | 0.069 | ) |
| POLG2_CpG_28.29.30 | 0.010 | ( | 0.000 | ) | 0.073 | ( | 0.006 | ) | 0.027 | ( | 0.006 | ) | 0.007 | ( | 0.006 | ) | 0.010 | ( | 0.000 | ) | 0.007 | ( | 0.006 | ) |
| POLG2_CpG_31.32 | 0.020 | ( | 0.000 | ) | 0.033 | ( | 0.006 | ) | 0.010 | ( | 0.000 | ) | 0.047 | ( | 0.006 | ) | 0.103 | ( | 0.006 | ) | 0.070 | ( | 0.052 | ) |
| POLG2_CpG_33.34 | 0.150 | ( | 0.017 | ) | 0.023 | ( | 0.006 | ) | 0.167 | ( | 0.012 | ) | 0.080 | ( | 0.056 | ) | 0.187 | ( | 0.012 | ) | 0.187 | ( | 0.012 | ) |
| POLG2_CpG_35.36 | 0.107 | ( | 0.006 | ) | 0.173 | ( | 0.006 | ) | 0.100 | ( | 0.010 | ) | 0.143 | ( | 0.064 | ) | 0.107 | ( | 0.006 | ) | 0.093 | ( | 0.029 | ) |
| POLG2_CpG_37.38.39.40 | 0.100 | ( | 0.000 | ) | 0.093 | ( | 0.006 | ) | 0.110 | ( | 0.017 | ) | 0.090 | ( | 0.010 | ) | 0.097 | ( | 0.006 | ) | 0.083 | ( | 0.006 | ) |
| POLG2_CpG_41 | 0.040 | ( | 0.017 | ) | 0.020 | ( | 0.000 | ) | 0.060 | ( | 0.010 | ) | 0.093 | ( | 0.006 | ) | 0.040 | ( | 0.000 | ) | 0.030 | ( | 0.017 | ) |
| TFAM_CpG_1 | 0.050 | ( | 0.000 | ) | 0.057 | ( | 0.006 | ) | 0.050 | ( | 0.020 | ) | 0.050 | ( | 0.010 | ) | 0.053 | ( | 0.006 | ) | 0.050 | ( | 0.000 | ) |
| TFAM_CpG_2.3 | 0.067 | ( | 0.006 | ) | 0.030 | ( | 0.020 | ) | 0.053 | ( | 0.032 | ) | 0.090 | ( | 0.010 | ) | 0.067 | ( | 0.006 | ) | 0.067 | ( | 0.006 | ) |
| TFAM_CpG_4 | 0.057 | ( | 0.006 | ) | 0.053 | ( | 0.006 | ) | 0.060 | ( | 0.010 | ) | 0.083 | ( | 0.006 | ) | 0.050 | ( | 0.017 | ) | 0.043 | ( | 0.029 | ) |
| TFAM_CpG_5 | 0.497 | ( | 0.049 | ) | 0.503 | ( | 0.006 | ) | 0.910 | ( | 0.056 | ) | 0.860 | ( | 0.056 | ) | 0.613 | ( | 0.012 | ) | 0.597 | ( | 0.015 | ) |
| TFAM_CpG_6 | 0.010 | ( | 0.000 | ) | 0.057 | ( | 0.029 | ) | 0.060 | ( | 0.010 | ) | 0.043 | ( | 0.029 | ) | 0.057 | ( | 0.006 | ) | 0.057 | ( | 0.006 | ) |
| TFAM_CpG_7 | 0.037 | ( | 0.015 | ) | 0.033 | ( | 0.029 | ) | 0.027 | ( | 0.025 | ) | 0.043 | ( | 0.006 | ) | 0.050 | ( | 0.000 | ) | 0.037 | ( | 0.023 | ) |
| TFAM_CpG_8 | 0.120 | ( | 0.106 | ) | 0.080 | ( | 0.000 | ) | 0.097 | ( | 0.091 | ) | 0.070 | ( | 0.000 | ) | 0.177 | ( | 0.029 | ) | 0.163 | ( | 0.006 | ) |
| TFAM_CpG_12 | 0.010 | ( | 0.000 | ) | 0.053 | ( | 0.046 | ) | 0.010 | ( | 0.000 | ) | 0.023 | ( | 0.006 | ) | 0.010 | ( | 0.000 | ) | 0.010 | ( | 0.000 | ) |
| FIS_1_CpG_1 | 0.010 | ( | 0.000 | ) | 0.060 | ( | 0.026 | ) | 0.017 | ( | 0.021 | ) | 0.040 | ( | 0.020 | ) | 0.023 | ( | 0.006 | ) | 0.020 | ( | 0.000 | ) |
| FIS_1_CpG_3 | 0.000 | ( | 0.000 | ) | 0.010 | ( | 0.000 | ) | 0.003 | ( | 0.006 | ) | 0.010 | ( | 0.000 | ) | 0.010 | ( | 0.000 | ) | 0.010 | ( | 0.000 | ) |
| FIS_1_CpG_4 | 0.060 | ( | 0.000 | ) | 0.060 | ( | 0.020 | ) | 0.070 | ( | 0.010 | ) | 0.067 | ( | 0.012 | ) | 0.077 | ( | 0.006 | ) | 0.080 | ( | 0.010 | ) |
| FIS_1_CpG_5 | 0.010 | ( | 0.000 | ) | 0.037 | ( | 0.006 | ) | 0.040 | ( | 0.035 | ) | 0.033 | ( | 0.015 | ) | 0.050 | ( | 0.000 | ) | 0.050 | ( | 0.000 | ) |
| FIS1_1_CpG_6 | 0.073 | ( | 0.006 | ) | 0.083 | ( | 0.021 | ) | 0.057 | ( | 0.015 | ) | 0.073 | ( | 0.042 | ) | 0.073 | ( | 0.006 | ) | 0.073 | ( | 0.006 | ) |
| FIS1_1_CpG_7.8 | 0.073 | ( | 0.012 | ) | 0.043 | ( | 0.006 | ) | 0.063 | ( | 0.021 | ) | 0.033 | ( | 0.006 | ) | 0.030 | ( | 0.000 | ) | 0.027 | ( | 0.006 | ) |
| FIS1_1_CpG_9 | 0.230 | ( | 0.000 | ) | 0.107 | ( | 0.021 | ) | 0.253 | ( | 0.166 | ) | 0.107 | ( | 0.012 | ) | 0.070 | ( | 0.000 | ) | 0.067 | ( | 0.006 | ) |
| FIS1_1_CpG_11 | 0.057 | ( | 0.006 | ) | 0.113 | ( | 0.057 | ) | 0.033 | ( | 0.025 | ) | 0.020 | ( | 0.017 | ) | 0.090 | ( | 0.000 | ) | 0.090 | ( | 0.000 | ) |
| FIS1_2_CpG_6 | 0.037 | ( | 0.006 | ) | 0.023 | ( | 0.012 | ) | 0.037 | ( | 0.012 | ) | 0.043 | ( | 0.025 | ) | 0.027 | ( | 0.006 | ) | 0.020 | ( | 0.000 | ) |
| FIS1_2_CpG_7 | 0.000 | ( | 0.000 | ) | 0.030 | ( | 0.000 | ) | 0.013 | ( | 0.023 | ) | 0.027 | ( | 0.006 | ) | 0.153 | ( | 0.214 | ) | 0.047 | ( | 0.006 | ) |
| FIS1_2_CpG_8 | 0.023 | ( | 0.006 | ) | 0.037 | ( | 0.040 | ) | 0.020 | ( | 0.026 | ) | 0.023 | ( | 0.012 | ) | 0.037 | ( | 0.006 | ) | 0.030 | ( | 0.000 | ) |
| FIS1_2_CpG_10 | 0.107 | ( | 0.012 | ) | 0.093 | ( | 0.025 | ) | 0.103 | ( | 0.006 | ) | 0.107 | ( | 0.006 | ) | 0.100 | ( | 0.000 | ) | 0.107 | ( | 0.012 | ) |
| FIS1_2_CpG_11.12.13 | 0.050 | ( | 0.000 | ) | 0.037 | ( | 0.006 | ) | 0.037 | ( | 0.006 | ) | 0.040 | ( | 0.020 | ) | 0.023 | ( | 0.006 | ) | 0.023 | ( | 0.006 | ) |
| FIS1_2_CpG_14.15 | 0.020 | ( | 0.000 | ) | 0.010 | ( | 0.000 | ) | 0.020 | ( | 0.000 | ) | 0.020 | ( | 0.000 | ) | 0.010 | ( | 0.000 | ) | 0.013 | ( | 0.006 | ) |
| FIS1_2_CpG_16.17.18 | 0.113 | ( | 0.012 | ) | 0.123 | ( | 0.021 | ) | 0.173 | ( | 0.015 | ) | 0.230 | ( | 0.056 | ) | 0.173 | ( | 0.012 | ) | 0.100 | ( | 0.010 | ) |
| OPA1_1_CpG_1 | 0.040 | ( | 0.000 | ) | 0.050 | ( | 0.000 | ) | 0.047 | ( | 0.006 | ) | 0.033 | ( | 0.006 | ) | 0.000 | ( | 0.000 | ) | 0.020 | ( | 0.010 | ) |
| OPA1_1_CpG_2 | 0.053 | ( | 0.006 | ) | 0.060 | ( | 0.046 | ) | 0.037 | ( | 0.006 | ) | 0.053 | ( | 0.015 | ) | 0.060 | ( | 0.010 | ) | 0.087 | ( | 0.015 | ) |
| OPA1_1_CpG_3 | 0.000 | ( | 0.000 | ) | 0.043 | ( | 0.012 | ) | 0.033 | ( | 0.006 | ) | 0.037 | ( | 0.006 | ) | 0.027 | ( | 0.006 | ) | 0.020 | ( | 0.010 | ) |
| OPA1_1_CpG_4 | 0.010 | ( | 0.000 | ) | 0.020 | ( | 0.010 | ) | 0.010 | ( | 0.000 | ) | 0.023 | ( | 0.006 | ) | 0.010 | ( | 0.000 | ) | 0.043 | ( | 0.021 | ) |
| OPA1_1_CpG_5.6 | 0.123 | ( | 0.006 | ) | 0.183 | ( | 0.140 | ) | 0.090 | ( | 0.017 | ) | 0.183 | ( | 0.137 | ) | 0.137 | ( | 0.012 | ) | 0.137 | ( | 0.072 | ) |
| OPA1_1_CpG_7 | 0.040 | ( | 0.000 | ) | 0.040 | ( | 0.010 | ) | 0.040 | ( | 0.000 | ) | 0.040 | ( | 0.010 | ) | 0.047 | ( | 0.006 | ) | 0.047 | ( | 0.006 | ) |
| OPA1_1_CpG_8 | 0.000 | ( | 0.000 | ) | 0.010 | ( | 0.000 | ) | 0.027 | ( | 0.006 | ) | 0.023 | ( | 0.006 | ) | 0.037 | ( | 0.006 | ) | 0.013 | ( | 0.006 | ) |
| OPA1_2_CpG_1 | 0.010 | ( | 0.000 | ) | 0.047 | ( | 0.006 | ) | 0.033 | ( | 0.006 | ) | 0.043 | ( | 0.012 | ) | 0.077 | ( | 0.006 | ) | 0.057 | ( | 0.049 | ) |
| OPA1_2_CpG_3 | 0.000 | ( | 0.000 | ) | 0.093 | ( | 0.049 | ) | 0.047 | ( | 0.015 | ) | 0.030 | ( | 0.010 | ) | 0.073 | ( | 0.006 | ) | 0.023 | ( | 0.015 | ) |
| OPA1_2_CpG_4 | 0.120 | ( | 0.000 | ) | 0.067 | ( | 0.060 | ) | 0.063 | ( | 0.031 | ) | 0.043 | ( | 0.015 | ) | 0.123 | ( | 0.012 | ) | 0.030 | ( | 0.026 | ) |
| OPA1_2_CpG_5 | 0.120 | ( | 0.000 | ) | 0.067 | ( | 0.060 | ) | 0.063 | ( | 0.031 | ) | 0.043 | ( | 0.015 | ) | 0.123 | ( | 0.012 | ) | 0.030 | ( | 0.026 | ) |
| OPA1_2_CpG_6 | 0.477 | ( | 0.021 | ) | 0.500 | ( | 0.010 | ) | 0.390 | ( | 0.125 | ) | 0.377 | ( | 0.055 | ) | 0.280 | ( | 0.017 | ) | 0.470 | ( | 0.026 | ) |
| OPA1_2_CpG_7 | 0.043 | ( | 0.006 | ) | 0.040 | ( | 0.000 | ) | 0.027 | ( | 0.006 | ) | 0.010 | ( | 0.010 | ) | 0.023 | ( | 0.006 | ) | 0.023 | ( | 0.021 | ) |
| OPA1_2_CpG_8 | 0.043 | ( | 0.006 | ) | 0.010 | ( | 0.000 | ) | 0.033 | ( | 0.006 | ) | 0.023 | ( | 0.021 | ) | 0.040 | ( | 0.000 | ) | 0.037 | ( | 0.015 | ) |
| OPA1_2_CpG_9 | 0.020 | ( | 0.000 | ) | 0.020 | ( | 0.000 | ) | 0.007 | ( | 0.006 | ) | 0.277 | ( | 0.006 | ) | 0.167 | ( | 0.006 | ) | 0.177 | ( | 0.006 | ) |
| OPA1_2_CpG_10.11 | 0.000 | ( | 0.000 | ) | 0.127 | ( | 0.040 | ) | 0.057 | ( | 0.015 | ) | 0.067 | ( | 0.055 | ) | 0.083 | ( | 0.006 | ) | 0.013 | ( | 0.006 | ) |
| OPA1_2_CpG_12 | 0.470 | ( | 0.010 | ) | 0.500 | ( | 0.010 | ) | 0.390 | ( | 0.125 | ) | 0.377 | ( | 0.055 | ) | 0.280 | ( | 0.017 | ) | 0.457 | ( | 0.015 | ) |
| OPA1_2_CpG_13 | 0.000 | ( | 0.000 | ) | 0.037 | ( | 0.006 | ) | 0.027 | ( | 0.006 | ) | 0.017 | ( | 0.006 | ) | 0.000 | ( | 0.000 | ) | 0.010 | ( | 0.000 | ) |
| OPA1_2_CpG_14.15 | 0.010 | ( | 0.000 | ) | 0.067 | ( | 0.015 | ) | 0.057 | ( | 0.015 | ) | 0.030 | ( | 0.010 | ) | 0.070 | ( | 0.000 | ) | 0.027 | ( | 0.021 | ) |
| OPA1_2_CpG_16 | 0.000 | ( | 0.000 | ) | 0.050 | ( | 0.000 | ) | 0.020 | ( | 0.010 | ) | 0.070 | ( | 0.010 | ) | 0.063 | ( | 0.006 | ) | 0.183 | ( | 0.038 | ) |
| OPA1_3_CpG_1 | 0.087 | ( | 0.006 | ) | 0.093 | ( | 0.050 | ) | 0.107 | ( | 0.021 | ) | 0.000 | ( | 0.000 | ) | 0.000 | ( | 0.000 | ) | 0.090 | ( | 0.010 | ) |
| OPA1_3_CpG_2.3 | 0.027 | ( | 0.006 | ) | 0.053 | ( | 0.006 | ) | 0.013 | ( | 0.015 | ) | 0.067 | ( | 0.035 | ) | 0.047 | ( | 0.006 | ) | 0.023 | ( | 0.021 | ) |
| OPA1_3_CpG_4 | 0.070 | ( | 0.000 | ) | 0.190 | ( | 0.010 | ) | 0.047 | ( | 0.012 | ) | 0.320 | ( | 0.026 | ) | 0.000 | ( | 0.000 | ) | 0.010 | ( | 0.000 | ) |
| OPA1_3_CpG_5 | 0.110 | ( | 0.000 | ) | 0.000 | ( | 0.000 | ) | 0.017 | ( | 0.006 | ) | 0.053 | ( | 0.006 | ) | 0.040 | ( | 0.000 | ) | 0.063 | ( | 0.032 | ) |
| OPA1_3_CpG_6.7 | 0.017 | ( | 0.006 | ) | 0.027 | ( | 0.006 | ) | 0.030 | ( | 0.000 | ) | 0.020 | ( | 0.010 | ) | 0.020 | ( | 0.000 | ) | 0.020 | ( | 0.010 | ) |
| OPA1_3_CpG_8.9 | 0.090 | ( | 0.010 | ) | 0.103 | ( | 0.015 | ) | 0.097 | ( | 0.064 | ) | 0.090 | ( | 0.000 | ) | 0.030 | ( | 0.000 | ) | 0.120 | ( | 0.010 | ) |
| OPA1_3_CpG_11.12 | 0.167 | ( | 0.202 | ) | 0.030 | ( | 0.035 | ) | 0.053 | ( | 0.006 | ) | 0.047 | ( | 0.031 | ) | 0.023 | ( | 0.006 | ) | 0.013 | ( | 0.012 | ) |
| OPA1_3_CpG_13.14.15.16 | 0.020 | ( | 0.010 | ) | 0.023 | ( | 0.015 | ) | 0.017 | ( | 0.006 | ) | 0.010 | ( | 0.000 | ) | 0.013 | ( | 0.006 | ) | 0.007 | ( | 0.006 | ) |
| OPA1_3_CpG_17 | 0.033 | ( | 0.006 | ) | 0.010 | ( | 0.000 | ) | 0.020 | ( | 0.010 | ) | 0.020 | ( | 0.010 | ) | 0.023 | ( | 0.006 | ) | 0.030 | ( | 0.017 | ) |
| OPA1_3_CpG_19 | 0.050 | ( | 0.010 | ) | 0.047 | ( | 0.012 | ) | 0.037 | ( | 0.012 | ) | 0.033 | ( | 0.015 | ) | 0.033 | ( | 0.006 | ) | 0.033 | ( | 0.006 | ) |
| OPA1_3_CpG_20 | 0.030 | ( | 0.010 | ) | 0.037 | ( | 0.006 | ) | 0.020 | ( | 0.000 | ) | 0.040 | ( | 0.020 | ) | 0.037 | ( | 0.006 | ) | 0.017 | ( | 0.015 | ) |
|  |  |  |  |  |  |  |  |  |  |  |  |  |  |  |  |  |  |  |  |  |  |  |  |  |
|  |  |  |  |  |  |  |  |  |  |  |  |  |  |  |  |  |  |  |  |  |  |  |  |  |
| **Kidney** | **27 weeks** | | | | | | | | **36 weeks** | | | | | | | | **96 weeks** | | | | | | | |
|  | **Standard diet** | | | | **Low-calorie diet** | | | | **Standard diet** | | | | **Low-calorie diet** | | | | **Standard diet** | | | | **Low-calorie diet** | | | |
|  | **Mean (SD)** | | | | **Mean (SD)** | | | | **Mean (SD)** | | | | **Mean (SD)** | | | | **Mean (SD)** | | | | **Mean (SD)** | | | |
| POLG_CpG_1 | 0.790 | ( | 0.139 | ) | 0.837 | ( | 0.023 | ) | 0.537 | ( | 0.215 | ) | 0.497 | ( | 0.215 | ) | 0.483 | ( | 0.012 | ) | 0.487 | ( | 0.006 | ) |
| POLG_CpG_2 | 0.700 | ( | 0.010 | ) | 0.490 | ( | 0.036 | ) | 0.590 | ( | 0.090 | ) | 0.533 | ( | 0.163 | ) | 0.603 | ( | 0.023 | ) | 0.667 | ( | 0.055 | ) |
| POLG_CpG_6.7 | 0.560 | ( | 0.010 | ) | 0.330 | ( | 0.000 | ) | 0.533 | ( | 0.021 | ) | 0.510 | ( | 0.026 | ) | 0.507 | ( | 0.064 | ) | 0.430 | ( | 0.017 | ) |
| POLG_CpG_9 | 0.407 | ( | 0.029 | ) | 0.300 | ( | 0.000 | ) | 0.380 | ( | 0.026 | ) | 0.400 | ( | 0.010 | ) | 0.513 | ( | 0.058 | ) | 0.537 | ( | 0.012 | ) |
| POLG_CpG_11.12 | 0.473 | ( | 0.023 | ) | 0.420 | ( | 0.020 | ) | 0.487 | ( | 0.012 | ) | 0.453 | ( | 0.015 | ) | 0.520 | ( | 0.069 | ) | 0.393 | ( | 0.012 | ) |
| POLG_CpG_13 | 0.760 | ( | 0.069 | ) | 0.610 | ( | 0.087 | ) | 0.777 | ( | 0.032 | ) | 0.517 | ( | 0.051 | ) | 0.823 | ( | 0.012 | ) | 0.510 | ( | 0.010 | ) |
| POLG_CpG_14 | 0.757 | ( | 0.029 | ) | 0.637 | ( | 0.064 | ) | 0.690 | ( | 0.030 | ) | 0.760 | ( | 0.010 | ) | 0.767 | ( | 0.006 | ) | 0.790 | ( | 0.026 | ) |
| POLG_CpG_15 | 0.613 | ( | 0.046 | ) | 0.510 | ( | 0.173 | ) | 0.500 | ( | 0.192 | ) | 0.747 | ( | 0.031 | ) | 0.447 | ( | 0.064 | ) | 0.723 | ( | 0.040 | ) |
| POLG2_CpG_1 | 0.000 | ( | 0.000 | ) | 0.500 | ( | 0.010 | ) | 0.030 | ( | 0.000 | ) | 0.523 | ( | 0.176 | ) | 0.040 | ( | 0.000 | ) | 0.517 | ( | 0.015 | ) |
| POLG2_CpG_2 | 0.087 | ( | 0.012 | ) | 0.040 | ( | 0.000 | ) | 0.010 | ( | 0.000 | ) | 0.103 | ( | 0.006 | ) | 0.053 | ( | 0.029 | ) | 0.003 | ( | 0.006 | ) |
| POLG2_CpG_3.4 | 0.123 | ( | 0.029 | ) | 0.077 | ( | 0.081 | ) | 0.167 | ( | 0.012 | ) | 0.163 | ( | 0.012 | ) | 0.093 | ( | 0.029 | ) | 0.057 | ( | 0.006 | ) |
| POLG2_CpG_5 | 0.313 | ( | 0.012 | ) | 0.117 | ( | 0.006 | ) | 0.313 | ( | 0.015 | ) | 0.303 | ( | 0.015 | ) | 0.280 | ( | 0.017 | ) | 0.077 | ( | 0.038 | ) |
| POLG2_CpG_6.7.8 | 0.200 | ( | 0.017 | ) | 0.243 | ( | 0.012 | ) | 0.243 | ( | 0.045 | ) | 0.233 | ( | 0.045 | ) | 0.240 | ( | 0.017 | ) | 0.260 | ( | 0.010 | ) |
| POLG2_CpG_9.10 | 0.093 | ( | 0.012 | ) | 0.260 | ( | 0.017 | ) | 0.103 | ( | 0.006 | ) | 0.157 | ( | 0.055 | ) | 0.077 | ( | 0.012 | ) | 0.220 | ( | 0.026 | ) |
| POLG2_CpG_11 | 0.070 | ( | 0.035 | ) | 0.093 | ( | 0.012 | ) | 0.097 | ( | 0.006 | ) | 0.100 | ( | 0.010 | ) | 0.053 | ( | 0.006 | ) | 0.083 | ( | 0.015 | ) |
| POLG2_CpG_12 | 0.233 | ( | 0.040 | ) | 0.057 | ( | 0.012 | ) | 0.203 | ( | 0.038 | ) | 0.067 | ( | 0.006 | ) | 0.307 | ( | 0.012 | ) | 0.047 | ( | 0.006 | ) |
| POLG2_CpG_13 | 0.277 | ( | 0.214 | ) | 0.000 | ( | 0.000 | ) | 0.293 | ( | 0.067 | ) | 0.287 | ( | 0.015 | ) | 0.030 | ( | 0.000 | ) | 0.557 | ( | 0.006 | ) |
| POLG2_CpG_14 | 0.060 | ( | 0.087 | ) | 0.073 | ( | 0.006 | ) | 0.033 | ( | 0.031 | ) | 0.200 | ( | 0.010 | ) | 0.147 | ( | 0.219 | ) | 0.067 | ( | 0.006 | ) |
| POLG2_CpG_15 | 0.010 | ( | 0.000 | ) | 0.107 | ( | 0.058 | ) | 0.030 | ( | 0.000 | ) | 0.067 | ( | 0.006 | ) | 0.010 | ( | 0.000 | ) | 0.070 | ( | 0.044 | ) |
| POLG2_CpG_16 | 0.017 | ( | 0.006 | ) | 0.090 | ( | 0.000 | ) | 0.033 | ( | 0.023 | ) | 0.073 | ( | 0.006 | ) | 0.013 | ( | 0.012 | ) | 0.013 | ( | 0.006 | ) |
| POLG2_CpG_17.18 | 0.220 | ( | 0.035 | ) | 0.093 | ( | 0.006 | ) | 0.257 | ( | 0.159 | ) | 0.417 | ( | 0.029 | ) | 0.170 | ( | 0.000 | ) | 0.163 | ( | 0.006 | ) |
| POLG2_CpG_19 | 0.010 | ( | 0.000 | ) | 0.193 | ( | 0.029 | ) | 0.000 | ( | 0.000 | ) | 0.283 | ( | 0.012 | ) | 0.000 | ( | 0.000 | ) | 0.233 | ( | 0.038 | ) |
| POLG2_CpG_20.21 | 0.220 | ( | 0.035 | ) | 0.107 | ( | 0.006 | ) | 0.257 | ( | 0.159 | ) | 0.443 | ( | 0.006 | ) | 0.170 | ( | 0.000 | ) | 0.007 | ( | 0.006 | ) |
| POLG2_CpG_22 | 0.113 | ( | 0.015 | ) | 0.193 | ( | 0.029 | ) | 0.107 | ( | 0.006 | ) | 0.283 | ( | 0.012 | ) | 0.000 | ( | 0.000 | ) | 0.233 | ( | 0.038 | ) |
| POLG2_CpG_23 | 0.113 | ( | 0.015 | ) | 0.010 | ( | 0.000 | ) | 0.223 | ( | 0.172 | ) | 0.153 | ( | 0.006 | ) | 0.033 | ( | 0.012 | ) | 0.037 | ( | 0.032 | ) |
| POLG2_CpG_24 | 0.133 | ( | 0.006 | ) | 0.010 | ( | 0.000 | ) | 0.107 | ( | 0.006 | ) | 0.147 | ( | 0.023 | ) | 0.010 | ( | 0.000 | ) | 0.177 | ( | 0.055 | ) |
| POLG2_CpG_25.26.27 | 0.107 | ( | 0.012 | ) | 0.010 | ( | 0.000 | ) | 0.033 | ( | 0.021 | ) | 0.010 | ( | 0.000 | ) | 0.063 | ( | 0.006 | ) | 0.037 | ( | 0.032 | ) |
| POLG2_CpG_28.29.30 | 0.043 | ( | 0.006 | ) | 0.000 | ( | 0.000 | ) | 0.060 | ( | 0.026 | ) | 0.030 | ( | 0.017 | ) | 0.057 | ( | 0.029 | ) | 0.033 | ( | 0.006 | ) |
| POLG2_CpG_31.32 | 0.000 | ( | 0.000 | ) | 0.020 | ( | 0.000 | ) | 0.013 | ( | 0.006 | ) | 0.030 | ( | 0.000 | ) | 0.013 | ( | 0.023 | ) | 0.097 | ( | 0.055 | ) |
| POLG2_CpG_33.34 | 0.160 | ( | 0.000 | ) | 0.007 | ( | 0.006 | ) | 0.183 | ( | 0.059 | ) | 0.063 | ( | 0.015 | ) | 0.153 | ( | 0.012 | ) | 0.013 | ( | 0.015 | ) |
| POLG2_CpG_35.36 | 0.093 | ( | 0.012 | ) | 0.177 | ( | 0.023 | ) | 0.103 | ( | 0.006 | ) | 0.230 | ( | 0.014 | ) | 0.077 | ( | 0.012 | ) | 0.197 | ( | 0.021 | ) |
| POLG2_CpG_37.38.39.40 | 0.100 | ( | 0.000 | ) | 0.093 | ( | 0.012 | ) | 0.097 | ( | 0.015 | ) | 0.100 | ( | 0.010 | ) | 0.097 | ( | 0.031 | ) | 0.083 | ( | 0.015 | ) |
| POLG2_CpG_41 | 0.043 | ( | 0.006 | ) | 0.020 | ( | 0.000 | ) | 0.037 | ( | 0.035 | ) | 0.020 | ( | 0.000 | ) | 0.063 | ( | 0.012 | ) | 0.023 | ( | 0.006 | ) |
| TFAM_CpG_1 | 0.033 | ( | 0.012 | ) | 0.053 | ( | 0.006 | ) | 0.050 | ( | 0.000 | ) | 0.063 | ( | 0.006 | ) | 0.053 | ( | 0.006 | ) | 0.057 | ( | 0.006 | ) |
| TFAM_CpG_2.3 | 0.097 | ( | 0.006 | ) | 0.037 | ( | 0.006 | ) | 0.107 | ( | 0.006 | ) | 0.103 | ( | 0.006 | ) | 0.060 | ( | 0.010 | ) | 0.063 | ( | 0.012 | ) |
| TFAM_CpG_4 | 0.070 | ( | 0.017 | ) | 0.050 | ( | 0.000 | ) | 0.080 | ( | 0.017 | ) | 0.067 | ( | 0.006 | ) | 0.077 | ( | 0.032 | ) | 0.090 | ( | 0.017 | ) |
| TFAM_CpG_5 | 0.597 | ( | 0.021 | ) | 0.600 | ( | 0.010 | ) | 0.603 | ( | 0.023 | ) | 0.543 | ( | 0.023 | ) | 0.693 | ( | 0.021 | ) | 0.703 | ( | 0.031 | ) |
| TFAM_CpG_6 | 0.050 | ( | 0.046 | ) | 0.043 | ( | 0.012 | ) | 0.087 | ( | 0.015 | ) | 0.060 | ( | 0.000 | ) | 0.077 | ( | 0.032 | ) | 0.077 | ( | 0.032 | ) |
| TFAM_CpG_7 | 0.010 | ( | 0.000 | ) | 0.053 | ( | 0.006 | ) | 0.010 | ( | 0.000 | ) | 0.040 | ( | 0.017 | ) | 0.060 | ( | 0.026 | ) | 0.067 | ( | 0.021 | ) |
| TFAM_CpG_8 | 0.327 | ( | 0.035 | ) | 0.153 | ( | 0.006 | ) | 0.383 | ( | 0.015 | ) | 0.310 | ( | 0.010 | ) | 0.563 | ( | 0.025 | ) | 0.577 | ( | 0.032 | ) |
| TFAM_CpG_12 | 0.000 | ( | 0.000 | ) | 0.013 | ( | 0.006 | ) | 0.010 | ( | 0.000 | ) | 0.010 | ( | 0.000 | ) | 0.057 | ( | 0.006 | ) | 0.047 | ( | 0.006 | ) |
| FIS_1_CpG_1 | 0.040 | ( | 0.035 | ) | 0.000 | ( | 0.000 | ) | 0.040 | ( | 0.000 | ) | 0.000 | ( | 0.000 | ) | 0.050 | ( | 0.000 | ) | 0.030 | ( | 0.000 | ) |
| FIS_1_CpG_3 | 0.000 | ( | 0.000 | ) | 0.110 | ( | 0.010 | ) | 0.010 | ( | 0.000 | ) | 0.010 | ( | 0.000 | ) | 0.000 | ( | 0.000 | ) | 0.020 | ( | 0.010 | ) |
| FIS_1_CpG_4 | 0.067 | ( | 0.012 | ) | 0.063 | ( | 0.006 | ) | 0.060 | ( | 0.010 | ) | 0.067 | ( | 0.006 | ) | 0.060 | ( | 0.000 | ) | 0.073 | ( | 0.006 | ) |
| FIS_1_CpG_5 | 0.007 | ( | 0.006 | ) | 0.030 | ( | 0.000 | ) | 0.010 | ( | 0.000 | ) | 0.020 | ( | 0.035 | ) | 0.057 | ( | 0.006 | ) | 0.010 | ( | 0.010 | ) |
| FIS1_1_CpG_6 | 0.083 | ( | 0.029 | ) | 0.080 | ( | 0.000 | ) | 0.080 | ( | 0.010 | ) | 0.083 | ( | 0.006 | ) | 0.040 | ( | 0.035 | ) | 0.067 | ( | 0.006 | ) |
| FIS1_1_CpG_7.8 | 0.053 | ( | 0.012 | ) | 0.037 | ( | 0.006 | ) | 0.047 | ( | 0.012 | ) | 0.037 | ( | 0.021 | ) | 0.027 | ( | 0.012 | ) | 0.020 | ( | 0.010 | ) |
| FIS1_1_CpG_9 | 0.143 | ( | 0.012 | ) | 0.020 | ( | 0.000 | ) | 0.117 | ( | 0.029 | ) | 0.240 | ( | 0.052 | ) | 0.070 | ( | 0.000 | ) | 0.070 | ( | 0.017 | ) |
| FIS1_1_CpG_11 | 0.063 | ( | 0.006 | ) | 0.043 | ( | 0.012 | ) | 0.057 | ( | 0.006 | ) | 0.060 | ( | 0.026 | ) | 0.047 | ( | 0.046 | ) | 0.070 | ( | 0.010 | ) |
| FIS1_2_CpG_6 | 0.033 | ( | 0.006 | ) | 0.037 | ( | 0.006 | ) | 0.037 | ( | 0.015 | ) | 0.030 | ( | 0.000 | ) | 0.030 | ( | 0.000 | ) | 0.003 | ( | 0.006 | ) |
| FIS1_2_CpG_7 | 0.027 | ( | 0.006 | ) | 0.013 | ( | 0.012 | ) | 0.047 | ( | 0.015 | ) | 0.023 | ( | 0.006 | ) | 0.053 | ( | 0.012 | ) | 0.020 | ( | 0.017 | ) |
| FIS1_2_CpG_8 | 0.007 | ( | 0.006 | ) | 0.107 | ( | 0.006 | ) | 0.000 | ( | 0.000 | ) | 0.000 | ( | 0.000 | ) | 0.000 | ( | 0.000 | ) | 0.060 | ( | 0.000 | ) |
| FIS1_2_CpG_10 | 0.083 | ( | 0.006 | ) | 0.097 | ( | 0.015 | ) | 0.087 | ( | 0.006 | ) | 0.097 | ( | 0.021 | ) | 0.103 | ( | 0.015 | ) | 0.070 | ( | 0.010 | ) |
| FIS1_2_CpG_11.12.13 | 0.023 | ( | 0.006 | ) | 0.033 | ( | 0.006 | ) | 0.037 | ( | 0.006 | ) | 0.023 | ( | 0.006 | ) | 0.030 | ( | 0.010 | ) | 0.037 | ( | 0.012 | ) |
| FIS1_2_CpG_14.15 | 0.020 | ( | 0.000 | ) | 0.017 | ( | 0.006 | ) | 0.023 | ( | 0.006 | ) | 0.023 | ( | 0.012 | ) | 0.017 | ( | 0.012 | ) | 0.030 | ( | 0.010 | ) |
| FIS1_2_CpG_16.17.18 | 0.120 | ( | 0.017 | ) | 0.193 | ( | 0.021 | ) | 0.100 | ( | 0.010 | ) | 0.110 | ( | 0.010 | ) | 0.113 | ( | 0.040 | ) | 0.097 | ( | 0.006 | ) |
| OPA1_1_CpG_1 | 0.023 | ( | 0.040 | ) | 0.027 | ( | 0.006 | ) | 0.067 | ( | 0.032 | ) | 0.050 | ( | 0.000 | ) | 0.027 | ( | 0.006 | ) | 0.047 | ( | 0.006 | ) |
| OPA1_1_CpG_2 | 0.043 | ( | 0.006 | ) | 0.053 | ( | 0.006 | ) | 0.040 | ( | 0.010 | ) | 0.050 | ( | 0.010 | ) | 0.040 | ( | 0.000 | ) | 0.077 | ( | 0.021 | ) |
| OPA1_1_CpG_3 | 0.037 | ( | 0.023 | ) | 0.033 | ( | 0.006 | ) | 0.050 | ( | 0.017 | ) | 0.020 | ( | 0.010 | ) | 0.020 | ( | 0.000 | ) | 0.037 | ( | 0.006 | ) |
| OPA1_1_CpG_4 | 0.043 | ( | 0.012 | ) | 0.023 | ( | 0.006 | ) | 0.040 | ( | 0.010 | ) | 0.010 | ( | 0.000 | ) | 0.033 | ( | 0.015 | ) | 0.037 | ( | 0.006 | ) |
| OPA1_1_CpG_5.6 | 0.097 | ( | 0.032 | ) | 0.157 | ( | 0.049 | ) | 0.107 | ( | 0.015 | ) | 0.117 | ( | 0.006 | ) | 0.073 | ( | 0.006 | ) | 0.170 | ( | 0.060 | ) |
| OPA1_1_CpG_7 | 0.057 | ( | 0.006 | ) | 0.043 | ( | 0.006 | ) | 0.070 | ( | 0.017 | ) | 0.053 | ( | 0.006 | ) | 0.057 | ( | 0.006 | ) | 0.057 | ( | 0.006 | ) |
| OPA1_1_CpG_8 | 0.037 | ( | 0.006 | ) | 0.023 | ( | 0.012 | ) | 0.010 | ( | 0.000 | ) | 0.017 | ( | 0.015 | ) | 0.010 | ( | 0.000 | ) | 0.010 | ( | 0.000 | ) |
| OPA1_2_CpG_1 | 0.010 | ( | 0.000 | ) | 0.033 | ( | 0.006 | ) | 0.040 | ( | 0.010 | ) | 0.043 | ( | 0.006 | ) | 0.010 | ( | 0.000 | ) | 0.000 | ( | 0.000 | ) |
| OPA1_2_CpG_3 | 0.027 | ( | 0.012 | ) | 0.083 | ( | 0.006 | ) | 0.033 | ( | 0.023 | ) | 0.067 | ( | 0.006 | ) | 0.000 | ( | 0.000 | ) | 0.037 | ( | 0.006 | ) |
| OPA1_2_CpG_4 | 0.087 | ( | 0.029 | ) | 0.050 | ( | 0.044 | ) | 0.083 | ( | 0.012 | ) | 0.007 | ( | 0.006 | ) | 0.080 | ( | 0.000 | ) | 0.097 | ( | 0.006 | ) |
| OPA1_2_CpG_5 | 0.090 | ( | 0.026 | ) | 0.050 | ( | 0.044 | ) | 0.083 | ( | 0.012 | ) | 0.007 | ( | 0.006 | ) | 0.087 | ( | 0.006 | ) | 0.097 | ( | 0.006 | ) |
| OPA1_2_CpG_6 | 0.487 | ( | 0.072 | ) | 0.477 | ( | 0.032 | ) | 0.453 | ( | 0.150 | ) | 0.333 | ( | 0.006 | ) | 0.527 | ( | 0.012 | ) | 0.577 | ( | 0.006 | ) |
| OPA1_2_CpG_7 | 0.020 | ( | 0.000 | ) | 0.040 | ( | 0.000 | ) | 0.013 | ( | 0.006 | ) | 0.007 | ( | 0.006 | ) | 0.010 | ( | 0.000 | ) | 0.010 | ( | 0.000 | ) |
| OPA1_2_CpG_8 | 0.027 | ( | 0.023 | ) | 0.093 | ( | 0.032 | ) | 0.010 | ( | 0.000 | ) | 0.103 | ( | 0.006 | ) | 0.000 | ( | 0.000 | ) | 0.097 | ( | 0.006 | ) |
| OPA1_2_CpG_9 | 0.020 | ( | 0.000 | ) | 0.043 | ( | 0.040 | ) | 0.010 | ( | 0.000 | ) | 0.010 | ( | 0.000 | ) | 0.053 | ( | 0.006 | ) | 0.017 | ( | 0.006 | ) |
| OPA1_2_CpG_10.11 | 0.063 | ( | 0.058 | ) | 0.097 | ( | 0.006 | ) | 0.037 | ( | 0.029 | ) | 0.077 | ( | 0.006 | ) | 0.200 | ( | 0.000 | ) | 0.047 | ( | 0.006 | ) |
| OPA1_2_CpG_12 | 0.490 | ( | 0.070 | ) | 0.477 | ( | 0.032 | ) | 0.467 | ( | 0.162 | ) | 0.333 | ( | 0.006 | ) | 0.523 | ( | 0.006 | ) | 0.577 | ( | 0.006 | ) |
| OPA1_2_CpG_13 | 0.000 | ( | 0.000 | ) | 0.013 | ( | 0.006 | ) | 0.000 | ( | 0.000 | ) | 0.007 | ( | 0.006 | ) | 0.013 | ( | 0.006 | ) | 0.077 | ( | 0.006 | ) |
| OPA1_2_CpG_14.15 | 0.076 | ( | 0.010 | ) | 0.023 | ( | 0.006 | ) | 0.037 | ( | 0.006 | ) | 0.023 | ( | 0.006 | ) | 0.020 | ( | 0.000 | ) | 0.027 | ( | 0.006 | ) |
| OPA1_2_CpG_16 | 0.077 | ( | 0.006 | ) | 0.117 | ( | 0.067 | ) | 0.040 | ( | 0.000 | ) | 0.083 | ( | 0.006 | ) | 0.147 | ( | 0.006 | ) | 0.010 | ( | 0.000 | ) |
| OPA1_3_CpG_1 | 0.023 | ( | 0.006 | ) | 0.000 | ( | 0.000 | ) | 0.080 | ( | 0.010 | ) | 0.077 | ( | 0.061 | ) | 0.083 | ( | 0.006 | ) | 0.077 | ( | 0.006 | ) |
| OPA1_3_CpG_2.3 | 0.030 | ( | 0.000 | ) | 0.057 | ( | 0.012 | ) | 0.037 | ( | 0.006 | ) | 0.013 | ( | 0.006 | ) | 0.030 | ( | 0.026 | ) | 0.050 | ( | 0.000 | ) |
| OPA1_3_CpG_4 | 0.070 | ( | 0.069 | ) | 0.083 | ( | 0.006 | ) | 0.090 | ( | 0.020 | ) | 0.063 | ( | 0.006 | ) | 0.047 | ( | 0.006 | ) | 0.100 | ( | 0.000 | ) |
| OPA1_3_CpG_5 | 0.027 | ( | 0.012 | ) | 0.053 | ( | 0.006 | ) | 0.080 | ( | 0.010 | ) | 0.013 | ( | 0.006 | ) | 0.033 | ( | 0.006 | ) | 0.077 | ( | 0.012 | ) |
| OPA1_3_CpG_6.7 | 0.023 | ( | 0.006 | ) | 0.023 | ( | 0.006 | ) | 0.027 | ( | 0.012 | ) | 0.027 | ( | 0.006 | ) | 0.037 | ( | 0.006 | ) | 0.077 | ( | 0.012 | ) |
| OPA1_3_CpG_8.9 | 0.063 | ( | 0.012 | ) | 0.107 | ( | 0.006 | ) | 0.077 | ( | 0.006 | ) | 0.170 | ( | 0.010 | ) | 0.140 | ( | 0.026 | ) | 0.117 | ( | 0.031 | ) |
| OPA1_3_CpG_11.12 | 0.030 | ( | 0.000 | ) | 0.020 | ( | 0.017 | ) | 0.037 | ( | 0.006 | ) | 0.057 | ( | 0.006 | ) | 0.030 | ( | 0.010 | ) | 0.010 | ( | 0.000 | ) |
| OPA1_3_CpG_13.14.15.16 | 0.030 | ( | 0.000 | ) | 0.023 | ( | 0.015 | ) | 0.030 | ( | 0.017 | ) | 0.013 | ( | 0.006 | ) | 0.027 | ( | 0.006 | ) | 0.027 | ( | 0.021 | ) |
| OPA1_3_CpG_17 | 0.020 | ( | 0.000 | ) | 0.033 | ( | 0.006 | ) | 0.020 | ( | 0.010 | ) | 0.020 | ( | 0.010 | ) | 0.057 | ( | 0.015 | ) | 0.023 | ( | 0.006 | ) |
| OPA1_3_CpG_19 | 0.033 | ( | 0.006 | ) | 0.053 | ( | 0.012 | ) | 0.040 | ( | 0.017 | ) | 0.043 | ( | 0.012 | ) | 0.043 | ( | 0.006 | ) | 0.037 | ( | 0.012 | ) |
| OPA1_3_CpG_20 | 0.020 | ( | 0.000 | ) | 0.047 | ( | 0.012 | ) | 0.027 | ( | 0.006 | ) | 0.020 | ( | 0.000 | ) | 0.023 | ( | 0.015 | ) | 0.063 | ( | 0.006 | ) |
|  |  |  |  |  |  |  |  |  |  |  |  |  |  |  |  |  |  |  |  |  |  |  |  |  |
|  |  |  |  |  |  |  |  |  |  |  |  |  |  |  |  |  |  |  |  |  |  |  |  |  |
|  |  |  |  |  |  |  |  |  |  |  |  |  |  |  |  |  |  |  |  |  |  |  |  |  |
| **Liver** | **27 weeks** | | | | | | | | **96 weeks** | | | | | | | | **36 weeks** | | | | | | | |
|  | **Standard diet** | | | | **Low-calorie diet** | | | | **Standard diet** | | | | **Low-calorie diet** | | | | **Standard diet** | | | | **Low-calorie diet** | | | |
|  | **Mean (SD)** | | | | **Mean (SD)** | | | | **Mean (SD)** | | | | **Mean (SD)** | | | | **Mean (SD)** | | | | **Mean (SD)** | | | |
| POLG_CpG_1 | 0.353 | ( | 0.035 | ) | 0.353 | ( | 0.032 | ) | 0.527 | ( | 0.032 | ) | 0.610 | ( | 0.017 | ) | 0.287 | ( | 0.006 | ) | 0.257 | ( | 0.006 | ) |
| POLG_CpG_2 | 0.353 | ( | 0.029 | ) | 0.467 | ( | 0.015 | ) | 0.313 | ( | 0.006 | ) | 0.333 | ( | 0.015 | ) | 0.313 | ( | 0.081 | ) | 0.323 | ( | 0.035 | ) |
| POLG_CpG_6.7 | 0.390 | ( | 0.020 | ) | 0.287 | ( | 0.015 | ) | 0.220 | ( | 0.017 | ) | 0.240 | ( | 0.017 | ) | 0.177 | ( | 0.040 | ) | 0.170 | ( | 0.017 | ) |
| POLG_CpG_9 | 0.433 | ( | 0.012 | ) | 0.433 | ( | 0.012 | ) | 0.153 | ( | 0.006 | ) | 0.140 | ( | 0.010 | ) | 0.103 | ( | 0.029 | ) | 0.103 | ( | 0.006 | ) |
| POLG_CpG_11.12 | 0.497 | ( | 0.006 | ) | 0.323 | ( | 0.006 | ) | 0.177 | ( | 0.006 | ) | 0.170 | ( | 0.010 | ) | 0.147 | ( | 0.023 | ) | 0.567 | ( | 0.015 | ) |
| POLG_CpG_13 | 0.793 | ( | 0.006 | ) | 0.343 | ( | 0.006 | ) | 0.223 | ( | 0.006 | ) | 0.213 | ( | 0.015 | ) | 0.160 | ( | 0.017 | ) | 0.573 | ( | 0.021 | ) |
| POLG_CpG_14 | 0.693 | ( | 0.006 | ) | 0.833 | ( | 0.006 | ) | 0.247 | ( | 0.015 | ) | 0.223 | ( | 0.025 | ) | 0.213 | ( | 0.012 | ) | 0.867 | ( | 0.006 | ) |
| POLG_CpG_15 | 0.487 | ( | 0.006 | ) | 0.557 | ( | 0.006 | ) | 0.207 | ( | 0.021 | ) | 0.203 | ( | 0.025 | ) | 0.213 | ( | 0.064 | ) | 0.823 | ( | 0.006 | ) |
| POLG2_CpG_1 | 0.000 | ( | 0.000 | ) | 0.307 | ( | 0.006 | ) | 0.037 | ( | 0.006 | ) | 0.017 | ( | 0.015 | ) | 0.023 | ( | 0.006 | ) | 0.573 | ( | 0.006 | ) |
| POLG2_CpG_2 | 0.000 | ( | 0.000 | ) | 0.083 | ( | 0.006 | ) | 0.000 | ( | 0.000 | ) | 0.100 | ( | 0.000 | ) | 0.030 | ( | 0.017 | ) | 0.010 | ( | 0.000 | ) |
| POLG2_CpG_3.4 | 0.067 | ( | 0.032 | ) | 0.023 | ( | 0.006 | ) | 0.093 | ( | 0.021 | ) | 0.100 | ( | 0.000 | ) | 0.037 | ( | 0.029 | ) | 0.063 | ( | 0.055 | ) |
| POLG2_CpG_5 | 0.237 | ( | 0.006 | ) | 0.140 | ( | 0.000 | ) | 0.243 | ( | 0.025 | ) | 0.167 | ( | 0.101 | ) | 0.307 | ( | 0.064 | ) | 0.053 | ( | 0.006 | ) |
| POLG2_CpG_6.7.8 | 0.207 | ( | 0.101 | ) | 0.277 | ( | 0.012 | ) | 0.267 | ( | 0.074 | ) | 0.207 | ( | 0.012 | ) | 0.220 | ( | 0.017 | ) | 0.010 | ( | 0.000 | ) |
| POLG2_CpG_9.10 | 0.130 | ( | 0.010 | ) | 0.297 | ( | 0.015 | ) | 0.093 | ( | 0.006 | ) | 0.133 | ( | 0.084 | ) | 0.087 | ( | 0.012 | ) | 0.177 | ( | 0.038 | ) |
| POLG2_CpG_11 | 0.107 | ( | 0.029 | ) | 0.083 | ( | 0.006 | ) | 0.057 | ( | 0.012 | ) | 0.067 | ( | 0.012 | ) | 0.110 | ( | 0.035 | ) | 0.087 | ( | 0.015 | ) |
| POLG2_CpG_12 | 0.237 | ( | 0.136 | ) | 0.017 | ( | 0.006 | ) | 0.130 | ( | 0.030 | ) | 0.067 | ( | 0.040 | ) | 0.133 | ( | 0.006 | ) | 0.093 | ( | 0.006 | ) |
| POLG2_CpG_13 | 0.010 | ( | 0.000 | ) | 0.490 | ( | 0.026 | ) | 0.013 | ( | 0.012 | ) | 0.010 | ( | 0.000 | ) | 0.063 | ( | 0.012 | ) | 0.520 | ( | 0.017 | ) |
| POLG2_CpG_14 | 0.100 | ( | 0.000 | ) | 0.160 | ( | 0.070 | ) | 0.050 | ( | 0.046 | ) | 0.037 | ( | 0.006 | ) | 0.037 | ( | 0.029 | ) | 0.010 | ( | 0.000 | ) |
| POLG2_CpG_15 | 0.000 | ( | 0.000 | ) | 0.067 | ( | 0.006 | ) | 0.100 | ( | 0.000 | ) | 0.097 | ( | 0.006 | ) | 0.073 | ( | 0.029 | ) | 0.010 | ( | 0.000 | ) |
| POLG2_CpG_16 | 0.000 | ( | 0.000 | ) | 0.010 | ( | 0.000 | ) | 0.000 | ( | 0.000 | ) | 0.093 | ( | 0.006 | ) | 0.073 | ( | 0.029 | ) | 0.080 | ( | 0.000 | ) |
| POLG2_CpG_17.18 | 0.173 | ( | 0.142 | ) | 0.083 | ( | 0.006 | ) | 0.230 | ( | 0.164 | ) | 0.040 | ( | 0.010 | ) | 0.363 | ( | 0.040 | ) | 0.343 | ( | 0.040 | ) |
| POLG2_CpG_19 | 0.007 | ( | 0.006 | ) | 0.140 | ( | 0.026 | ) | 0.007 | ( | 0.006 | ) | 0.340 | ( | 0.572 | ) | 0.000 | ( | 0.000 | ) | 0.280 | ( | 0.000 | ) |
| POLG2_CpG_20.21 | 0.227 | ( | 0.067 | ) | 0.170 | ( | 0.000 | ) | 0.230 | ( | 0.164 | ) | 0.037 | ( | 0.006 | ) | 0.363 | ( | 0.040 | ) | 0.377 | ( | 0.021 | ) |
| POLG2_CpG_22 | 0.200 | ( | 0.010 | ) | 0.140 | ( | 0.026 | ) | 0.033 | ( | 0.029 | ) | 0.000 | ( | 0.000 | ) | 0.130 | ( | 0.017 | ) | 0.277 | ( | 0.006 | ) |
| POLG2_CpG_23 | 0.120 | ( | 0.026 | ) | 0.087 | ( | 0.006 | ) | 0.017 | ( | 0.015 | ) | 0.013 | ( | 0.006 | ) | 0.227 | ( | 0.081 | ) | 0.173 | ( | 0.006 | ) |
| POLG2_CpG_24 | 0.013 | ( | 0.006 | ) | 0.047 | ( | 0.006 | ) | 0.053 | ( | 0.006 | ) | 0.050 | ( | 0.000 | ) | 0.127 | ( | 0.015 | ) | 0.083 | ( | 0.006 | ) |
| POLG2_CpG_25.26.27 | 0.010 | ( | 0.000 | ) | 0.043 | ( | 0.006 | ) | 0.010 | ( | 0.010 | ) | 0.010 | ( | 0.000 | ) | 0.000 | ( | 0.000 | ) | 0.010 | ( | 0.000 | ) |
| POLG2_CpG_28.29.30 | 0.000 | ( | 0.000 | ) | 0.060 | ( | 0.000 | ) | 0.023 | ( | 0.012 | ) | 0.010 | ( | 0.000 | ) | 0.027 | ( | 0.012 | ) | 0.057 | ( | 0.006 | ) |
| POLG2_CpG_31.32 | 0.023 | ( | 0.006 | ) | 0.020 | ( | 0.000 | ) | 0.060 | ( | 0.026 | ) | 0.013 | ( | 0.006 | ) | 0.060 | ( | 0.035 | ) | 0.013 | ( | 0.006 | ) |
| POLG2_CpG_33.34 | 0.207 | ( | 0.101 | ) | 0.013 | ( | 0.006 | ) | 0.123 | ( | 0.055 | ) | 0.087 | ( | 0.032 | ) | 0.230 | ( | 0.035 | ) | 0.027 | ( | 0.015 | ) |
| POLG2_CpG_35.36 | 0.133 | ( | 0.006 | ) | 0.177 | ( | 0.021 | ) | 0.093 | ( | 0.006 | ) | 0.120 | ( | 0.053 | ) | 0.087 | ( | 0.012 | ) | 0.113 | ( | 0.032 | ) |
| POLG2_CpG_37.38.39.40 | 0.100 | ( | 0.000 | ) | 0.083 | ( | 0.006 | ) | 0.100 | ( | 0.000 | ) | 0.093 | ( | 0.012 | ) | 0.100 | ( | 0.000 | ) | 0.073 | ( | 0.015 | ) |
| POLG2_CpG_41 | 0.050 | ( | 0.000 | ) | 0.020 | ( | 0.000 | ) | 0.037 | ( | 0.012 | ) | 0.020 | ( | 0.000 | ) | 0.033 | ( | 0.023 | ) | 0.017 | ( | 0.006 | ) |
| TFAM_CpG_1 | 0.050 | ( | 0.000 | ) | 0.063 | ( | 0.006 | ) | 0.040 | ( | 0.010 | ) | 0.077 | ( | 0.006 | ) | 0.057 | ( | 0.040 | ) | 0.040 | ( | 0.010 | ) |
| TFAM_CpG_2.3 | 0.050 | ( | 0.000 | ) | 0.050 | ( | 0.000 | ) | 0.063 | ( | 0.038 | ) | 0.033 | ( | 0.006 | ) | 0.130 | ( | 0.010 | ) | 0.133 | ( | 0.015 | ) |
| TFAM_CpG_4 | 0.040 | ( | 0.000 | ) | 0.050 | ( | 0.000 | ) | 0.080 | ( | 0.010 | ) | 0.090 | ( | 0.000 | ) | 0.047 | ( | 0.023 | ) | 0.040 | ( | 0.026 | ) |
| TFAM_CpG_5 | 0.563 | ( | 0.012 | ) | 0.600 | ( | 0.010 | ) | 0.737 | ( | 0.021 | ) | 0.960 | ( | 0.053 | ) | 0.613 | ( | 0.015 | ) | 0.630 | ( | 0.010 | ) |
| TFAM_CpG_6 | 0.040 | ( | 0.000 | ) | 0.010 | ( | 0.000 | ) | 0.080 | ( | 0.010 | ) | 0.087 | ( | 0.006 | ) | 0.047 | ( | 0.023 | ) | 0.040 | ( | 0.026 | ) |
| TFAM_CpG_7 | 0.130 | ( | 0.000 | ) | 0.193 | ( | 0.006 | ) | 0.047 | ( | 0.015 | ) | 0.053 | ( | 0.006 | ) | 0.097 | ( | 0.015 | ) | 0.103 | ( | 0.015 | ) |
| TFAM_CpG_8 | 0.230 | ( | 0.000 | ) | 0.230 | ( | 0.000 | ) | 0.113 | ( | 0.006 | ) | 0.103 | ( | 0.006 | ) | 0.343 | ( | 0.012 | ) | 0.350 | ( | 0.010 | ) |
| TFAM_CpG_12 | 0.000 | ( | 0.000 | ) | 0.020 | ( | 0.000 | ) | 0.010 | ( | 0.000 | ) | 0.010 | ( | 0.000 | ) | 0.030 | ( | 0.000 | ) | 0.063 | ( | 0.006 | ) |
| FIS_1_CpG_1 | 0.040 | ( | 0.000 | ) | 0.023 | ( | 0.015 | ) | 0.047 | ( | 0.015 | ) | 0.067 | ( | 0.006 | ) | 0.050 | ( | 0.000 | ) | 0.027 | ( | 0.006 | ) |
| FIS_1_CpG_3 | 0.000 | ( | 0.000 | ) | 0.103 | ( | 0.006 | ) | 0.000 | ( | 0.000 | ) | 0.010 | ( | 0.000 | ) | 0.000 | ( | 0.000 | ) | 0.010 | ( | 0.000 | ) |
| FIS_1_CpG_4 | 0.050 | ( | 0.017 | ) | 0.067 | ( | 0.006 | ) | 0.057 | ( | 0.006 | ) | 0.067 | ( | 0.015 | ) | 0.057 | ( | 0.006 | ) | 0.053 | ( | 0.012 | ) |
| FIS_1_CpG_5 | 0.000 | ( | 0.000 | ) | 0.007 | ( | 0.006 | ) | 0.010 | ( | 0.000 | ) | 0.010 | ( | 0.000 | ) | 0.050 | ( | 0.000 | ) | 0.000 | ( | 0.000 | ) |
| FIS1_1_CpG_6 | 0.093 | ( | 0.012 | ) | 0.077 | ( | 0.031 | ) | 0.000 | ( | 0.000 | ) | 0.073 | ( | 0.029 | ) | 0.050 | ( | 0.000 | ) | 0.073 | ( | 0.012 | ) |
| FIS1_1_CpG_7.8 | 0.063 | ( | 0.006 | ) | 0.040 | ( | 0.020 | ) | 0.050 | ( | 0.010 | ) | 0.060 | ( | 0.036 | ) | 0.033 | ( | 0.006 | ) | 0.037 | ( | 0.006 | ) |
| FIS1_1_CpG_9 | 0.000 | ( | 0.000 | ) | 0.233 | ( | 0.204 | ) | 0.220 | ( | 0.026 | ) | 0.207 | ( | 0.021 | ) | 0.103 | ( | 0.006 | ) | 0.000 | ( | 0.000 | ) |
| FIS1_1_CpG_11 | 0.070 | ( | 0.000 | ) | 0.030 | ( | 0.026 | ) | 0.090 | ( | 0.010 | ) | 0.000 | ( | 0.000 | ) | 0.070 | ( | 0.000 | ) | 0.100 | ( | 0.017 | ) |
| FIS1_2_CpG_6 | 0.040 | ( | 0.010 | ) | 0.027 | ( | 0.006 | ) | 0.040 | ( | 0.010 | ) | 0.037 | ( | 0.012 | ) | 0.060 | ( | 0.010 | ) | 0.040 | ( | 0.000 | ) |
| FIS1_2_CpG_7 | 0.043 | ( | 0.012 | ) | 0.033 | ( | 0.015 | ) | 0.047 | ( | 0.031 | ) | 0.000 | ( | 0.000 | ) | 0.010 | ( | 0.000 | ) | 0.037 | ( | 0.006 | ) |
| FIS1_2_CpG_8 | 0.007 | ( | 0.006 | ) | 0.020 | ( | 0.000 | ) | 0.000 | ( | 0.000 | ) | 0.027 | ( | 0.006 | ) | 0.023 | ( | 0.006 | ) | 0.117 | ( | 0.159 | ) |
| FIS1_2_CpG_10 | 0.103 | ( | 0.012 | ) | 0.093 | ( | 0.006 | ) | 0.087 | ( | 0.021 | ) | 0.090 | ( | 0.017 | ) | 0.107 | ( | 0.021 | ) | 0.107 | ( | 0.023 | ) |
| FIS1_2_CpG_11.12.13 | 0.037 | ( | 0.012 | ) | 0.033 | ( | 0.006 | ) | 0.040 | ( | 0.035 | ) | 0.047 | ( | 0.006 | ) | 0.033 | ( | 0.015 | ) | 0.037 | ( | 0.012 | ) |
| FIS1_2_CpG_14.15 | 0.013 | ( | 0.006 | ) | 0.013 | ( | 0.006 | ) | 0.010 | ( | 0.000 | ) | 0.017 | ( | 0.006 | ) | 0.020 | ( | 0.010 | ) | 0.013 | ( | 0.006 | ) |
| FIS1_2_CpG_16.17.18 | 0.150 | ( | 0.010 | ) | 0.130 | ( | 0.035 | ) | 0.143 | ( | 0.031 | ) | 0.120 | ( | 0.010 | ) | 0.143 | ( | 0.032 | ) | 0.170 | ( | 0.030 | ) |
| OPA1_1_CpG_1 | 0.057 | ( | 0.029 | ) | 0.100 | ( | 0.010 | ) | 0.160 | ( | 0.010 | ) | 0.077 | ( | 0.006 | ) | 0.137 | ( | 0.084 | ) | 0.101 | ( | 0.009 | ) |
| OPA1_1_CpG_2 | 0.047 | ( | 0.006 | ) | 0.060 | ( | 0.020 | ) | 0.040 | ( | 0.010 | ) | 0.057 | ( | 0.006 | ) | 0.060 | ( | 0.010 | ) | 0.043 | ( | 0.006 | ) |
| OPA1_1_CpG_3 | 0.040 | ( | 0.035 | ) | 0.070 | ( | 0.056 | ) | 0.013 | ( | 0.006 | ) | 0.040 | ( | 0.000 | ) | 0.030 | ( | 0.010 | ) | 0.053 | ( | 0.006 | ) |
| OPA1_1_CpG_4 | 0.023 | ( | 0.021 | ) | 0.040 | ( | 0.000 | ) | 0.010 | ( | 0.000 | ) | 0.207 | ( | 0.012 | ) | 0.027 | ( | 0.006 | ) | 0.040 | ( | 0.017 | ) |
| OPA1_1_CpG_5.6 | 0.110 | ( | 0.010 | ) | 0.110 | ( | 0.020 | ) | 0.127 | ( | 0.032 | ) | 0.143 | ( | 0.015 | ) | 0.147 | ( | 0.032 | ) | 0.057 | ( | 0.006 | ) |
| OPA1_1_CpG_7 | 0.037 | ( | 0.006 | ) | 0.060 | ( | 0.010 | ) | 0.020 | ( | 0.010 | ) | 0.037 | ( | 0.006 | ) | 0.040 | ( | 0.010 | ) | 0.040 | ( | 0.010 | ) |
| OPA1_1_CpG_8 | 0.030 | ( | 0.000 | ) | 0.030 | ( | 0.030 | ) | 0.017 | ( | 0.006 | ) | 0.030 | ( | 0.000 | ) | 0.020 | ( | 0.010 | ) | 0.013 | ( | 0.006 | ) |
| OPA1_2_CpG_1 | 0.010 | ( | 0.000 | ) | 0.047 | ( | 0.006 | ) | 0.047 | ( | 0.006 | ) | 0.047 | ( | 0.006 | ) | 0.010 | ( | 0.000 | ) | 0.000 | ( | 0.000 | ) |
| OPA1_2_CpG_3 | 0.013 | ( | 0.006 | ) | 0.027 | ( | 0.006 | ) | 0.033 | ( | 0.006 | ) | 0.010 | ( | 0.000 | ) | 0.047 | ( | 0.029 | ) | 0.030 | ( | 0.000 | ) |
| OPA1_2_CpG_4 | 0.067 | ( | 0.023 | ) | 0.020 | ( | 0.010 | ) | 0.007 | ( | 0.006 | ) | 0.187 | ( | 0.006 | ) | 0.017 | ( | 0.012 | ) | 0.060 | ( | 0.000 | ) |
| OPA1_2_CpG_5 | 0.070 | ( | 0.026 | ) | 0.020 | ( | 0.010 | ) | 0.007 | ( | 0.006 | ) | 0.187 | ( | 0.006 | ) | 0.017 | ( | 0.012 | ) | 0.060 | ( | 0.000 | ) |
| OPA1_2_CpG_6 | 0.433 | ( | 0.021 | ) | 0.340 | ( | 0.095 | ) | 0.573 | ( | 0.012 | ) | 0.557 | ( | 0.006 | ) | 0.710 | ( | 0.010 | ) | 0.490 | ( | 0.082 | ) |
| OPA1_2_CpG_7 | 0.020 | ( | 0.000 | ) | 0.027 | ( | 0.006 | ) | 0.010 | ( | 0.010 | ) | 0.000 | ( | 0.000 | ) | 0.017 | ( | 0.006 | ) | 0.020 | ( | 0.000 | ) |
| OPA1_2_CpG_8 | 0.010 | ( | 0.000 | ) | 0.017 | ( | 0.012 | ) | 0.067 | ( | 0.006 | ) | 0.010 | ( | 0.000 | ) | 0.040 | ( | 0.000 | ) | 0.010 | ( | 0.000 | ) |
| OPA1_2_CpG_9 | 0.020 | ( | 0.000 | ) | 0.013 | ( | 0.006 | ) | 0.007 | ( | 0.006 | ) | 0.010 | ( | 0.000 | ) | 0.017 | ( | 0.006 | ) | 0.020 | ( | 0.000 | ) |
| OPA1_2_CpG_10.11 | 0.043 | ( | 0.006 | ) | 0.037 | ( | 0.006 | ) | 0.203 | ( | 0.006 | ) | 0.010 | ( | 0.000 | ) | 0.010 | ( | 0.000 | ) | 0.203 | ( | 0.006 | ) |
| OPA1_2_CpG_12 | 0.427 | ( | 0.015 | ) | 0.340 | ( | 0.095 | ) | 0.563 | ( | 0.006 | ) | 0.557 | ( | 0.006 | ) | 0.703 | ( | 0.006 | ) | 0.490 | ( | 0.082 | ) |
| OPA1_2_CpG_13 | 0.000 | ( | 0.000 | ) | 0.023 | ( | 0.006 | ) | 0.007 | ( | 0.006 | ) | 0.010 | ( | 0.000 | ) | 0.010 | ( | 0.000 | ) | 0.000 | ( | 0.000 | ) |
| OPA1_2_CpG_14.15 | 0.043 | ( | 0.006 | ) | 0.017 | ( | 0.006 | ) | 0.010 | ( | 0.000 | ) | 0.010 | ( | 0.000 | ) | 0.017 | ( | 0.006 | ) | 0.033 | ( | 0.012 | ) |
| OPA1_2_CpG_16 | 0.010 | ( | 0.000 | ) | 0.010 | ( | 0.000 | ) | 0.007 | ( | 0.006 | ) | 0.010 | ( | 0.000 | ) | 0.127 | ( | 0.006 | ) | 0.123 | ( | 0.006 | ) |
| OPA1_3_CpG_1 | 0.000 | ( | 0.000 | ) | 0.053 | ( | 0.006 | ) | 0.060 | ( | 0.053 | ) | 0.050 | ( | 0.000 | ) | 0.103 | ( | 0.006 | ) | 0.020 | ( | 0.035 | ) |
| OPA1_3_CpG_2.3 | 0.073 | ( | 0.006 | ) | 0.053 | ( | 0.012 | ) | 0.043 | ( | 0.006 | ) | 0.063 | ( | 0.006 | ) | 0.043 | ( | 0.021 | ) | 0.060 | ( | 0.030 | ) |
| OPA1_3_CpG_4 | 0.000 | ( | 0.000 | ) | 0.200 | ( | 0.010 | ) | 0.037 | ( | 0.006 | ) | 0.057 | ( | 0.006 | ) | 0.097 | ( | 0.021 | ) | 0.613 | ( | 0.023 | ) |
| OPA1_3_CpG_5 | 0.050 | ( | 0.010 | ) | 0.033 | ( | 0.012 | ) | 0.053 | ( | 0.015 | ) | 0.040 | ( | 0.010 | ) | 0.053 | ( | 0.006 | ) | 0.057 | ( | 0.032 | ) |
| OPA1_3_CpG_6.7 | 0.017 | ( | 0.006 | ) | 0.033 | ( | 0.006 | ) | 0.027 | ( | 0.006 | ) | 0.023 | ( | 0.015 | ) | 0.033 | ( | 0.006 | ) | 0.020 | ( | 0.000 | ) |
| OPA1_3_CpG_8.9 | 0.053 | ( | 0.006 | ) | 0.200 | ( | 0.030 | ) | 0.120 | ( | 0.046 | ) | 0.117 | ( | 0.021 | ) | 0.053 | ( | 0.049 | ) | 0.050 | ( | 0.010 | ) |
| OPA1_3_CpG_11.12 | 0.023 | ( | 0.015 | ) | 0.027 | ( | 0.006 | ) | 0.010 | ( | 0.000 | ) | 0.040 | ( | 0.010 | ) | 0.017 | ( | 0.006 | ) | 0.020 | ( | 0.010 | ) |
| OPA1_3_CpG_13.14.15.16 | 0.023 | ( | 0.006 | ) | 0.020 | ( | 0.010 | ) | 0.027 | ( | 0.015 | ) | 0.027 | ( | 0.015 | ) | 0.013 | ( | 0.006 | ) | 0.010 | ( | 0.000 | ) |
| OPA1_3_CpG_17 | 0.037 | ( | 0.006 | ) | 0.010 | ( | 0.000 | ) | 0.047 | ( | 0.015 | ) | 0.023 | ( | 0.006 | ) | 0.040 | ( | 0.010 | ) | 0.010 | ( | 0.000 | ) |
| OPA1_3_CpG_19 | 0.057 | ( | 0.006 | ) | 0.027 | ( | 0.012 | ) | 0.063 | ( | 0.015 | ) | 0.040 | ( | 0.010 | ) | 0.047 | ( | 0.006 | ) | 0.037 | ( | 0.015 | ) |
| OPA1_3_CpG_20 | 0.057 | ( | 0.006 | ) | 0.037 | ( | 0.006 | ) | 0.010 | ( | 0.000 | ) | 0.047 | ( | 0.006 | ) | 0.033 | ( | 0.021 | ) | 0.060 | ( | 0.010 | ) |
